# Facilitators and barriers to enhancing physical activity in older patients during acute hospital stay: a systematic review

**DOI:** 10.1186/s12966-022-01330-z

**Published:** 2022-07-30

**Authors:** F. Dijkstra, G. van der Sluis, H. Jager-Wittenaar, L. Hempenius, J. S. M. Hobbelen, E. Finnema

**Affiliations:** 1 Research Group Living, Wellbeing and Care for Older People, NHL Stenden University of Applied Sciences, Rengerslaan 8-10, P.O. Box 1080, 8900, CB Leeuwarden, The Netherlands; 2 Research Group Healthy Ageing, Allied Health Care and Nursing, Hanze University of Applied Sciences, Groningen, The Netherlands; 3 Department of Health Science, Section of Nursing Research & Education, University Medical Center Groningen, University of Groningen, Groningen, The Netherlands; 4 FAITH research, Groningen/Leeuwarden, The Netherlands; 5 Department of Health Strategy and Innovation, Nij Smellinghe Hospital Drachten, Drachten, The Netherlands; 6 Department of Oral and Maxillofacial Surgery, University Medical Center Groningen, University of Groningen, Groningen, The Netherlands; 7 Geriatric Center, Medical Center Leeuwarden, Leeuwarden, The Netherlands; 8 Department of General Practice and Elderly Care Medicine, University Medical Center Groningen, University of Groningen, Groningen, The Netherlands; 9 Research Group Nursing Diagnostics, Hanze University of Applied Sciences, Groningen, The Netherlands

**Keywords:** Physical activity, Hospital, Older adults, Facilitator, Barrier, Systematic review

## Abstract

**Background:**

To improve older patients’ physical activity (PA) behavior, it is important to identify facilitators and barriers to enhancing PA in older patients (≥ 65 years) during hospitalization from the perspectives of patients, caregivers, and healthcare professionals (HCPs).

**Methods:**

In this systematic review, a search of PubMed, CINAHL, PsycINFO, EMBASE, and Web of Science (January 2000–May 2021) was performed, and quantitative, qualitative, and mixed-methods studies were included. The methodological quality of included studies was assessed using the Mixed Methods Appraisal Tool. Identified facilitators and barriers were categorized using the social ecological model at the intrapersonal, interpersonal, and institutional levels.

**Results:**

The 48 included articles identified 230 facilitators and 342 barriers. The main facilitators at the intrapersonal level included: knowledge, awareness, and attitudes; interpersonal level: social support, including encouragement and interdisciplinary collaboration; and institutional level: stimulating physical environment, patient activities and schedules, and PA protocols. The main barriers at the intrapersonal level included: physical health status, having lines or drains, patients’ fear, and HCPs’ safety concerns; interpersonal level: patient-HCP relation and HCPs’ unclear roles; and institutional level: lack of space and resources, including time and equipment. Best evidence synthesis provided moderate level of evidence for three barriers: patients’ unwillingness or refusal to move, patients having symptoms, and patients having lines or drains. No moderate level of evidence was found for facilitators.

**Conclusion:**

The PA behavior of older adults during hospitalization is multidimensional. Our overview highlights facilitators and barriers on multilevel scale (intrapersonal, interpersonal, and institutional levels) that guides patients, caregivers, HCPs, and researchers in future clinical practice, and intervention development and implementation.

**Supplementary Information:**

The online version contains supplementary material available at 10.1186/s12966-022-01330-z.

## Introduction

Aging is associated with a higher prevalence of age-related diseases and health-related events [1]. Figures from high income countries show that up to 50% of all patients admitted to the hospital are over 65 years [2–4], which is expected to increase in the coming years with an increasingly older population [1]. Regardless of disease on admission or treatment, hospitalized older patients are at increased risk of loss of functional capacity, which can be long lasting, even after discharge [5–8]. Loss of functional capacity is defined as the loss of ability to perform activities of daily living (ADL), which may lead to considerable consequences, including increased risk of readmission, institutionalization, and even death [9–12]. Low in-hospital physical activity (PA) behavior is an important risk factor for loss of functional capacity that can be prevented [5, 6, 8, 10, 13].

Although PA is important to prevent loss of functional capacity in older patients, few patients engage in PA during hospital admission. Figures show that older patients, including patients who might be able to ambulate independently, spend at least 80% of the day lying in bed during hospitalization [14–17]. Restricted PA and bedrest are harmful, resulting in large reductions in muscle mass and function, loss of aerobic capacity, increase in fatigue, and decrease in quality of life [18–22]. Especially for older patients, the impact of bed rest and low PA appears to have more deleterious effects on the aforementioned muscle function, aerobic capacity, and ADL functioning than for younger adults [18, 22, 23].

It is indicated that augmenting PA during hospitalization seems to be effective for improving physical functioning, emotional status, social well-being [24], and may lead to a possible reduction in patient falls [25]. However, despite the proven added value of PA, the idea of ‘being cared for’ and ‘lying in the hospital’ seems deeply rooted and should change into ‘working on recovery’ in order to increase patients’ PA behavior [7, 26]. To achieve this change, it would be necessary to gain insight into factors that positively or negatively contribute to the level of PA in older patients during hospitalization.

To the best of our knowledge, a systematic overview of the literature regarding facilitators and barriers toward PA in older hospitalized patients is lacking. Recently, studies identifying facilitators or barriers to enhancing PA have been reviewed for hospitalized patients of all ages [27–29], as well as for older patients in the home setting [30, 31]. From these home-setting studies, it is known that there might be specific factors for older persons, such as fear of falling, that are barriers to PA [30]. Furthermore, it is known that there are specific hospital-setting factors, such as the influence of healthcare professionals (HCPs), patients’ health status or lack of resources [27–29]. Given the specific hospital setting in the current review, in which patients enter a different and unfamiliar context, it is conceivable that facilitators and barriers might be different for patients over 65 years than for younger patients.

The first step in changing PA behavior in older hospitalized patients is to explore which modifiable factors are related to PA. The aim of this study was to systematically review the literature focusing on the identification of facilitators and barriers to enhance PA in older patients (≥ 65 y) during hospital admission from the perspectives of patients, caregivers, and HCPs (e.g., nurses, physiotherapists, medical doctors, dietitians) working in an acute care setting with older patients.

## Method

This systematic review followed the Preferred Reporting Items for Systematic Reviews and Meta-Analyses (PRISMA) guidelines (Additional files 1 and 2) [32]. The protocol was registered in PROSPERO (CRD42020172923).

### Criteria for inclusion and exclusion

To be included in this review, studies had to explore facilitators and/or barriers to PA in older patients during hospitalization. Studies were included if: 1) they were published between January 1, 2000, and May 29, 2021; 2) they were written in English, German, or Dutch; 3) they used a quantitative, qualitative, or mixed-methods study design; and 4) the study population consisted of patients aged ≥65 years (baseline mean age of 65 years or older), caregivers, or HCPs. Furthermore, PA was defined as any kind of bodily movement produced by skeletal muscles that results in energy expenditure [33]. Therefore, for this review, PA concerned all patient activities (e.g., ADL, with or without assistance sitting, standing, walking, or exercising) that contributed to preventing functional decline. Barriers were defined as perceived obstacles to engage in behavior to enhance PA, and facilitators were defined as the forces acting in, on, or around a person to encourage PA behavior.

Studies were excluded if they were: 1) editorials, opinion papers, conference abstracts, or literature reviews; 2) targeting patients with moderate to severe dementia according to standard diagnostic criteria (e.g., DSM-4-TR, DSM-5, NINCDS-ADRDA, and NINCDS-AIREN) or palliative patients; 3) studies with no original participant data regarding barriers and facilitators; and 4) studies that were performed in an inpatient rehabilitation or psychiatric unit, or in healthcare settings other than hospitals, such as outpatient clinics.

### Search strategy

A literature search was conducted using PubMed, CINAHL, PsycINFO, EMBASE, and Web of Science databases (Additional file 3). The search string combined the following key words: hospitalization, PA, facilitators or barriers, and older patients. For each keyword, synonyms and subject heading terms were included. The search strategy was drafted with the help of an experienced librarian.

All records obtained were deduplicated using RefWorks bibliographic management software (ProQuest). The titles and abstracts of the retrieved studies were independently screened by two reviewers (FD and GS) using Rayyan [34]. After initial screening, the full texts of these studies were retrieved and independently screened for eligibility. In case of discrepancies, these were resolved by discussion and, with remaining doubts, by seeking the opinion of another reviewer (HH).

### Quality assessment

Two reviewers (FD and GS) independently assessed the methodological quality of each included study using the validated Mixed Methods Appraisal Tool (MMAT, version 2018) [35]. The MMAT is a critical appraisal tool that is designed for appraisal of systematic mixed studies reviews, i.e. reviews that include qualitative, quantitative and/or mixed methods studies [35]. The MMAT was chosen to appraise a diverse study design types. The use of one instrument, instead of using a diverse set of instruments for different study design types, provided the opportunity to compare methodological quality results with each other.

The MMAT has five sets of criteria for the following five categories: a) qualitative, b) randomized controlled trials (RCTs), c) non-randomized, d) quantitative descriptive, and e) mixed-methods studies. For each included study, the appropriate set of criteria was rated on a categorical scale (yes, no, cannot tell). For mixed-method studies, 15 criteria were rated instead of five. Discrepancies were discussed until consensus was reached, and when necessary, they were resolved with a third reviewer (HH).

To provide an indication of the quality of each study (low or high), an overall quality score was calculated. The overall quality score reflected the number of criteria satisfied, varying from no criteria met to all five criteria met. For mixed-method studies, the lowest score was used as overall quality score. Subsequently, the overall quality scores of each study were used to group studies into two categories: high (MMAT score 4 or 5) and low (MMAT score 0–3) quality. The categories were determined based on a previous similar review [36] and by team consensus. An overall quality score was preferred instead of a detailed presentation of the ratings as suggested by the MMAT authors [35], since these categories were not used as inclusion or exclusion criteria.

### Data extraction and synthesis

One researcher (FD) extracted data from the selected full-text studies. The characteristics extracted included author details, year of publication, aim, study design, methods, participant characteristics, and main results. For the facilitators and barriers, the original study had to indicate whether a factor was a facilitator or barrier to enhancing PA. Uncertainties or questionable issues were resolved by discussion between two authors (FD, HH). A randomly selected 20% of the data extraction records were cross-checked by another author (GS).

Data synthesis was performed by two researchers (FD and GS). A framework synthesis was used as a starting point to synthesize the factors influencing PA, and the integration of qualitative and quantitative research emerged [37, 38]. The adapted framework of the social ecological model [39] was therefore used, consisting of intrapersonal, interpersonal, and institutional levels (Additional file 4), which was used previously in systematic reviews [30, 31]. The extracted facilitators and/or barriers with related concepts were organized into descriptive major themes and subthemes using an inductive approach. Next, (sub)themes were categorized based on the levels of the social ecological model. Furthermore, to determine whether reported PA interventions were possibly working as facilitators or barriers to PA, the characteristics of the performed PA intervention and their clinical effects were described. Per included study, the whole PA intervention was interpreted as one factor that might enhance patients’ PA, since it was impossible to distinguish what kind of element within the PA intervention was the facilitating and/or hampering factor.

### Best evidence synthesis

The level of evidence of facilitators and/or barriers from quantitative studies and quantitative parts of mixed-methods studies was assessed using the best evidence synthesis approach [40, 41]. The approach was applied to facilitators and barriers that were reported at least once in RCTs or at least twice in non-randomized studies. The approach considered the study’s quality (defined by MMAT scores), study design, the number of articles, and finding consistency, to classify the evidence as strong, moderate, limited, conflicting, or insufficient [36] (Table 1).

**Table 1:** Best evidence synthesis guidelines

Level of evidence	Explanation
Strong	Consistent findings^a^ in two or more RCTs of high quality (MMAT score 4 or 5).
Moderate	Consistent findings in one high-quality RCT and one or more RCTs of low quality (MMAT score 0–3), or one high-quality RCT and one or more high-quality non-randomized studies.
Limited	Consistent findings in one high-quality RCT, or two or more high-quality non-randomized studies, or one or more high-quality non-randomized studies, and one or more low-quality RCTs.
Conflicting	contradictory findings, with less than 75% of the studies reporting consistent findings.
Insufficient	The above criteria were not met.

^a^ Results were considered consistent when ≥75% of the studies demonstrated findings in the same direction

## Results

### Study selection and characteristics

**Fig. 1:**
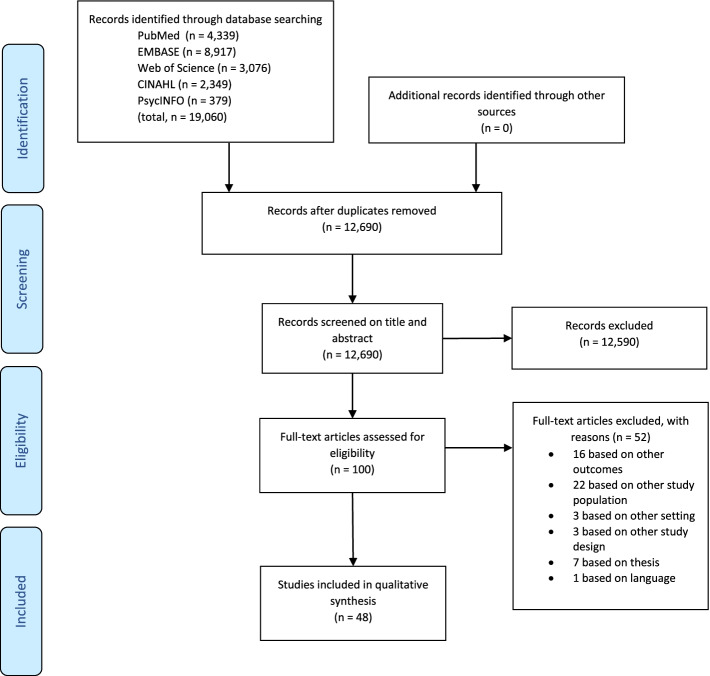
PRISMA flow diagram of each stage of the study selection

A flowchart of the search strategy is presented in Fig. 1. The initial search of the databases yielded 19,060 records. After removing duplicates, a total of 12,690 articles were screened on title and abstract, of which 12,590 did not meet the inclusion criteria. Of the 100 articles for which the full text was read, another 52 did not meet the inclusion criteria. The 48 articles included in the review comprised 18 qualitative, 19 quantitative, and 11 mixed-methods studies (Additional file 5). The studies were conducted in 14 countries, of which the most common were the USA (*n* = 16) and Australia (*n* = 6). Over half (60%) of the included studies have been published since 2018. Twenty studies focused on patient perspectives [42–61], ten described HCPs’ perspectives [62–71], 16 focused on both patients and HCPs [72–86], one described patient and caregiver perspectives [87], and one described patient, caregiver, and HCP perspectives [88]. Furthermore, 12 studies [49, 51, 53, 56, 72, 74, 76–80, 83] investigated the effects of a PA intervention by comparing two study population groups.

### Quality assessment of included studies

The methodological quality scores of the included quantitative studies ranged from no score to a maximum score of 5 (Additional file 6). Of the 19 quantitative studies, 13 studies were categorized as low quality [42, 46–49, 53, 55, 57, 58, 64, 69, 73, 74]. Six quantitative studies were categorized as high quality [44, 45, 50, 51, 56, 85]. The methodological quality of qualitative studies ranged from a score of 3 to 5, of which three studies were categorized as low quality [59, 68, 87], and 15 studies were categorized as high quality [43, 52, 54, 61–63, 65–67, 71, 81, 82, 86, 88, 89]. For the mixed-methods studies, the total methodological quality scores varied from no score to a score of 2, with all 11 studies categorized as low quality [60, 70, 72, 75–80, 83, 84].

### Facilitators and barriers

Of the 48 articles, 230 facilitators and 342 barriers related to PA in older hospitalized patients were retrieved. These factors were reduced to a total of 17 major themes and 39 subthemes across the three perspectives (Table 2). Examples of facilitators and barriers are provided to reflect themes. Our best evidence synthesis results ranged from insufficient to moderate levels of evidence, of which results classified with limited and moderate levels of evidence were reported in the sections below. Further details of the unique facilitators and barriers mapped to the (sub)themes are presented in Additional file 7, and the best evidence synthesis classification is presented in Additional file 8.

**Table 2:** Themes and subthemes influencing PA in older hospitalized patients within the social-ecological model

Social ecological model	Theme	Subtheme	Facilitator^**a**^		Barrier^**a**^	
			Quantitative^**b**^	Qualitative^**c**^	Quantitative^**b**^	Qualitative^**c**^
**Patient perspective**						
Intrapersonal level	1. Patient knowledge, awareness, and attitude	1.1 Knowledge, awareness, and attitude	8	24	6	19
	2. Patients personal and health factors	2.1 Patient personal factors	2	0	5	0
		2.2 Emotional status	1	1	5	9
		2.3 Cognitive status	1	0	2	0
		2.4 Physical health	8	0	12	14
	3. Medical-related factors	3.1 Presence of lines/attachments	1	0	4	5
		3.2 Admitting diagnosis and illness severity	4	0	3	1
		3.3 Treatment-related factors	3	0	3	2
Interpersonal level	4. Social support	4.1 Patient - informal network	0	5	3	1
		4.2 Patient - HCP	3	13	1	18
Institutional level	5. Physical environment	5.1 Space and location	2	6	3	7
	6. Resources	6.1 Staffing	0	0	0	4
		6.2 Time and competing priorities	0	0	0	2
		6.3 Equipment	0	0	0	2
		6.4 Education and information	0	1	0	1
	7. Organizational factors	7.1 Hospital routines and activities	3	8	2	4
		7.2 Daytime or weekday	2	0	5	0
		7.3 Rules, regulations and policies	0	1	0	1
**Caregiver perspective**						
Intrapersonal level	8. Caregiver knowledge, awareness, and attitude	8.1 Knowledge, awareness, and attitude	n/a	3	n/a	4
		8.2 Patient safety concerns	n/a	0	n/a	2
Interpersonal level	9. Patients health status and medical-related factors	9.1 Physical or mental health	n/a	0	n/a	2
Institutional level	n/a		n/a	0	n/a	0
**Healthcare professional perspective**						
Intrapersonal level	10. HCP knowledge, awareness, and attitude	10.1 Knowledge, awareness, and attitude	4	11	5	15
		10.2 Patient safety concerns	0	0	5	3
	11. HCP expertise and characteristics	11.1 Expertise and characteristics	1	4	5	1
Interpersonal level	12. Patient cooperation	12.1 Patient - informal network	0	5	0	3
		12.2 Patient - HCP	1	15	3	27
	13. Clinician and team influences	13.1 Collaboration	1	14	1	4
		13.2 Role clarity	2	3	0	8
		13.3 Responsibility	2	7	0	8
	14. Patients health status and medical-related factors	14.1 Physical or mental health	1	3	8	12
		14.2 Treatment-related factors	1	3	0	7
Institutional level	15. Physical environment	15.1 Space and location	0	9	0	9
	16. Resources	16.1 Staffing	1	4	2	8
		16.2 Time and competing priorities	0	2	8	19
		16.3 Equipment	1	6	1	5
		16.4 Education and information	0	7	2	1
		16.5 Monitoring and documentation	0	5	0	3
	17. Organizational factors	17.1 Hospital routines and activities	0	9	0	7
		17.2 Rules, regulations and policies	0	8	0	10

Described by the three perspectives of patient, caregiver and healthcare professional

*n/a* not applicable, *HCP* healthcare professionals

^a^ = Number of items according to the dimensions of the Social-Ecological Model, themes and subthemes

^b^ = Number of items retrieved from quantitative studies

^c^ = Number of items retrieved from qualitative studies

#### Patient perspective

##### Intrapersonal level

In total, 53 facilitators and 90 barriers at the intrapersonal level were identified, resulting in theme 1) patients’ knowledge, awareness, and attitude; theme 2) patients’ personal and health factors; and theme 3) medical-related factors.

Common facilitators of PA of theme 1 ‘patients’ knowledge, awareness and attitude’ were: patients’ positive attitudes, or expectations toward mobilization [52, 61, 76, 80, 81]. Furthermore, patients’ awareness of the need for mobility [59, 60, 88, 89], patients who want to promote functional recovery [54, 59, 77, 84], and perceived benefits due to mobilization were facilitators [52, 54, 60, 76, 87]. Facilitators of theme 2 ‘physical health’ were: sufficient baseline functional status scores, or sufficient baseline PA level of patients [42, 50, 55, 57, 84, 85]. Facilitators of theme 3 ‘medical-related factors’ were: improving medical status or no acute complications [45, 50], and lower illness severity scores [55, 57].

Common barriers to PA on theme 1 ‘patients’ knowledge, awareness, and attitude’ were negative attitudes of patients [52, 76, 80]. Specifically, patients’ expectation that hospital admission was not associated with mobilization hampered PA [81]. Furthermore, patients refused to move [49, 51], or they preferred to stay in bed, because they believed it would contribute to aiding recovery [60, 80, 81, 88]. Barriers of theme 2 ‘physical health’ were: patients’ fears, in particular fear of falling [43, 51, 60, 82, 84, 86, 88], fear of burden to nurse when falling [88], and fear of injury [51, 54, 59, 84]. Furthermore, frequently reported barrier was patients with poor or impaired physical health status [52, 81, 86, 89]. Specifically, patients having symptoms such as pain, dyspnea, dizziness, feeling ill, or fatigue [42, 43, 48, 51, 54, 60, 61, 76, 82, 84, 85, 87]. On theme 3 ‘medical-related factors’ was the presence of lines or attachments that hindered patients walking a barrier, such as catheters [45, 50, 51, 54, 59, 60, 81, 82].

The best evidence synthesis suggested moderate evidence for three barriers: patients’ unwillingness or refusal to move, patients having symptoms, and having lines or drains (Additional file 8). Limited evidence was found for two facilitators: higher baseline functional status score and improved medical status, and two barriers: patients’ fears and continuous oxygen therapy.

##### Interpersonal level

At the interpersonal level, 21 facilitators and 23 barriers were identified, resulting in theme 4) social support. Facilitators of theme 4 ‘social support’ were: the identification and support of family or friends [43, 61, 76, 87], and the presence and encouragement of professionals to enhance mobilization [43, 54, 60, 61, 75, 89].

Common barriers of theme 4 ‘social support’ were: patients being alone and lacking encouragement [51, 75], and the presence of visitors whereby patients were sitting in bed [75, 89]. In addition, HCPs’ discouragement or lack of support [51, 54, 59, 89] and lack of interest in the importance of mobility [82] were barriers. Furthermore, patients felt that they were not seen or known by HCPs [43, 89], and they received inconsistent or limited advice on mobility [59, 86] or how to handle tubes while walking [59].

The best evidence synthesis suggested limited evidence for two barriers: lack of companion encouragement and lack of professional help.

##### Institutional level

In total, 23 facilitators and 31 barriers were identified and were reduced to theme 5) physical environment, theme 6) resources, and theme 7) organizational factors.

Common facilitators of theme 5 ‘physical environment’ were: more space in patient rooms, ambulation routes, and seating and handrails in hallways [43, 53, 81, 83, 89]. A facilitator of theme 6 ‘resources’ was staff education to understand patients [43]. Facilitators for theme 7 ‘organizational factors’ were daily schedules [43, 81], patient goal setting [52, 87], and hospital routines and providing ADL, particularly in the morning, with statistically significant improvement of PA [75].

Barriers of theme 5 ‘physical environment’ were: uninviting furnishing in the department, clutter in rooms or hallways, limited seating [59, 61], and lack of space to mobilize [51]. Barriers of theme 6 ‘resources’ were: staff shortage or staff lacking time [61, 82, 88], and lack of equipment and assistive devices [51, 54, 60]. Barriers to theme 7 ‘organizational factors’ were the first day of admission [42, 48, 50] and the afternoon [75].

The best evidence synthesis suggested limited evidence for three barriers: lack of space, lack of equipment, and first day of admission.

#### Caregiver perspective

##### Intrapersonal level

Three facilitators and six barriers at the intrapersonal level were identified in two studies, resulting in theme 8) caregivers’ knowledge, awareness, and attitude. Facilitators of theme 8 were: awareness of the need for mobility and seeing benefits of mobilization [87, 88]. Barriers to theme 8 were anxiety or feeling overwhelmed [87], perceived bedrest to aid recovery [88], and safety concerns regarding patient falling [88].

##### Interpersonal level

Zero facilitators and two barriers of patient mobilization were identified at the interpersonal level, resulting in theme 9) patients’ health status and medical-related factors. Barriers reported in one study were poor patient physical symptoms, such as pain or fatigue, and comorbidities [87].

##### Institutional level

No facilitators and barriers were identified at the institutional level.

#### Health care professional

##### Intrapersonal level

In total, 20 facilitators and 34 barriers were identified, resulting in theme 10) HCPs’ knowledge, awareness, and attitude, and theme 11) HCPs’ expertise and characteristics.

Common facilitators of theme 10 ‘HCPs’ knowledge, awareness, and attitude’ were HCPs experiencing positive benefits of PA for themselves and patients [76, 77], and awareness of mobility importance [71, 84, 88, 90]. Facilitators of theme 11 ‘HCPs’ expertise and characteristics’ were experience in geriatric specialization, long-term care setting, or rehabilitation [65, 67].

Common barriers of theme 10 ‘HCPs’ knowledge, awareness, and attitude’ were not seeing PA as part of usual hospital care or questioning the need for PA for older patients, as specifically reported by nurses [65, 70, 81]. Furthermore, barriers were staff resistance toward health promotion [66, 89], or staff lacking awareness of the intervention [76]. Moreover, and commonly reported, HCPs lacked knowledge, including mobilization techniques [62, 68], patient mobility status and safety assessment [68, 78, 80], patients’ psychosocial needs, and how to inform or motivate them [62, 81]. In addition, on theme 10, barriers were fear of patient falling [62, 82] and safety concerns regarding staff or patient injury [64, 68, 69, 85].

##### Interpersonal level

In total, 58 facilitators and 81 barriers were identified, resulting in theme 12) patient cooperation, theme 13) clinician and team influences, and theme 14) patient health status and medical-related factors.

Common facilitators of theme 12 ‘patient cooperation’ were: family support and involvement of patient’s family in care provision to enhance PA [62, 63, 65, 67, 88], HCP encouragement [62, 65, 67, 70, 71], HCPs knowing patients and their functional ability [62, 63], and physiotherapists in ‘sporty clothes’ [67]. Facilitators of theme 13 ‘clinician and team influences’ were: multidisciplinary collaboration [62, 63, 66, 71, 78, 80, 89] and being responsible for patient mobility [72, 79, 90], including shifting responsibility toward nurses.

Common barriers of theme 12 ‘patient cooperation’ were: patients lacking knowledge and awareness [68, 90], the adoption of a sick-role behavior of patients, including wearing pajamas during the day [63, 67, 68, 70, 90], and rejection of patients to mobilize [64, 68, 85], especially when asked by nurses [67]. In addition, on theme 12, barriers were: the belief of family that patients needed to rest in bed [62], quiet or nursing home patients [62, 65], and during contact with patients, HCPs told unintentionally how comfortable the bed was or HCPs were servicing self-supporting patients [67, 70, 81].

Common barriers of theme 13 ‘clinician and team influences’ were: discussion or lack of communication between staff [68, 78, 80], unclear HCPs’ roles, and lack of professional autonomy toward patients’ mobilization [62, 66, 86]. In addition, on theme 13, HCPs defined mobilization differently, including which tasks and actions were important to influence patients’ mobility [67, 80]. Furthermore, nurses and physicians did not perceive mobilization as part of their core tasks [14, 67]. Barriers on theme 14 ‘patients health status and medical-related factors’ were: patients’ poor health condition [62–65, 67, 71, 73, 82, 85, 86], the presence of lines or attachments [68, 81, 82], and bed rest orders [63, 65, 76].

##### Institutional level

In total, 52 facilitators and 75 barriers at the institutional level were identified, resulting in theme 15) physical environment, theme 16) resources, and theme 17) organizational factors.

The facilitator of theme 15 ‘physical environment’ was an encouraging hospital environment [62, 67, 80, 81, 83, 88], including space for walking. Facilitators of theme 16 ‘resources’ were: presence of mobility equipment [62, 65, 68, 70], patient education [62, 68], staff training [66, 68, 80], and monitoring patients’ mobility status [65, 68, 79]. Facilitators of theme 17 ‘organizational factors’ were: setting patient goals [72, 79], providing daily patient activities [68, 81, 89], and integrating PA into daily usual care [66, 72]. In addition, on theme 17, facilitators were facility-wide adoption of PA promoting philosophy, clear expectations of unit level and accountability, and alignment with institutional priorities to improve patient mobilization [62, 65, 66, 70].

Common barriers of theme 15 ‘physical environment’ were lack of space, intimidating environment, or objects in corridors [62, 67, 68, 76, 78, 82]. Barriers of theme 16 ‘resources’ were: shortage of staff, especially in the evening and during weekend [62, 64, 67, 68, 73, 82, 88], workload [63, 64, 68, 70, 73], and time and competing priorities of staff leading to lower prioritization of PA [49, 62–64, 67, 68, 70, 71, 76, 78, 80–82, 85, 86, 88, 89]. In addition, on theme 16, barriers were: lack of proper mobility equipment [68, 78, 80, 82], and lack of system to document and monitor mobility [68, 80]. Barriers of theme 17 ‘organizational factors’ were busy days [65, 76], lack of follow-through on mobilization [62], and staff starting ambulation in phase ‘getting ready for discharge’ with little time [65]. Furthermore, barriers of theme 17 were unfamiliarity and lack of mobility protocols [80, 81], and policies deterring PA, including zero-fall initiatives [62, 63, 80, 86, 88].

The best evidence synthesis suggested limited evidence for one barrier: time constraints.

### Studies with PA intervention as a possible facilitator

Twelve quantitative studies described a PA intervention and reported clinical intervention effects (Table 3). In total, four types of PA interventions were identified that might act as facilitators to increase patients’ PA. Facilitating factors were: 1) adding extra patient ambulation sessions provided by physiotherapists [78], volunteers [76], or mobility technicians [49]; 2) changing the physical environment, including more space in patient rooms to mobilize [53, 83]; 3) adding a tool in daily patient practice to enhance patient PA, including a booklet with PA information [51] or a patient’s activity board [56]; and 4) PA interventions with more than one changing element, for example, a combination of the aforementioned facilitating factors of PA interventions, or a combination of these with training of HCPs in communication or collaboration [72, 74, 77, 79, 80].

**Table 3:** Type of PA intervention (versus control) with clinical intervention effects (*n* = 11)

First author, year of publication	Type of intervention versus control	Main results^**a**^
Feenstra et al., 2021 [83]	Reactivating hospital concept with 8 hours of patient activation, 8 hours of relaxation, and 8 hours of sleep. Interventions included 1) room turned into a studio with a living room area, 2) niches in corridors with own theme (see, hear, write, and exercise) to activate patients, and 3) on department level, a meeting room, a relaxation room, and a garden room were provided; versus usual care pre-intervention.	↓ Lying in bed*, ↑ Sitting, ↑ Walking
Hamilton et al., 2019 [49]	Three times daily assisted ambulation by mobility technicians (under supervision of physiotherapist); versus not seen by mobility technician (usual care)	↑ Step count/day, ↑ Patients achieved ≥400 and ≥ 900 step goal/day, ↑ Basic mobility from admission to discharge, ↑ Length of stay
King et al., 2016 [72]	MOVIN intervention. Five elements: 1) Psychomotor skills training, 2) Improve communication between HCPs, 3) Ambulation pathways and visual markers, 4) Increase ambulation resources, 5) Ambulation culture; versus usual care preintervention	↑ Total ambulation frequency/week*, ↑ Total ambulation distance/week*, ↓ Nursing staff numeric documentation
Moreno et al., 2019 [51]	Booklet with content about the deleterious effects of hospitalization and the importance of staying active during hospitalization; versus no booklet (usual care)	↑ Step count/day, ↓ Mobility loss from admission to discharge, ↑ Light intensive PA, ↑ Moderate intensity PA, ↓ Sedentary time
Mudge et al., 2015 [77]	Eat Walk Engage program aiming: 1) support adequate nutritional intake, 2) Promote early exercise and ambulation, 3) Provide therapeutic activities to reduce complications; versus monthly audit implementation data	↑ Nursing documentation on (target domains) cognitive status, mobility assistance requirements, nutritional assistance, = Nursing documentation on (target domain) level of recommended activity, ↑ Patient self-reported target activities (sat out in chair, gone for a walk, activity to keep mind active), ↓ Length of stay
Porserud et al., 2019 [56]	Activity board with daily goals on mobilization set by physiotherapist and patient; versus standard treatment	↑ Step count/day*, ↓ Lying in bed*, ↑ Upright (standing + walking)*, ↑ Standing*, ↑ Walking*, ↑ Total upright (sitting + standing + walking)*, ↑ Sitting, ↑ Transitions from sit to stand*, ↓ Length of stay*, ↑ Bowel function (first flatus, first stool)*
Resnick et al., 2015 [74]	FFC-AC intervention. Three components: 1) Nurses’ education and training, 2) Environment and policy assessment, 3) Ongoing training and motivation of nurses; versus nurses’ education only (FFC-ED).	= Nurses’ mean scores on Knowledge Function Focused Care test
S. Lim et al., 2020 [76]	Twice daily volunteer-led mobility or bedside exercises; versus on average twice-weekly seen by therapist (usual care)	↑ Step count/day, ↓ Length of stay, ↓ 30-day hospital readmission
Shannon et al., 2019 [53]	New ward with 1) more single patient-rooms, 2) family space in room, 3) family lounge and interview room, 4) ‘wrap around’ corridor and 5) therapy room; versus old ward with only family space in single room, one lounge room (for family and staff), linear corridor, and no therapy room.	↑ PA out of bed*, ↑ PA at bedside (< 1 m)*, ↑ PA at patient bay (> 1 m, < 3 m of bed)*, ↓ Patient social activity in bed*, ↑ Patient social activity at bedside (< 1 m)*, ↑ Patient social activity in patient bay (> 1 m, < 3 m of bed)*
Tousignant-Laflamme et al., 2015 [78]	Adding physiotherapy services in the emergency department with an individualized intervention plan per patient, continued when admitted to the ward; versus patients who did not received physiotherapy treatment on the emergency department (usual care).	↓ Immobilization syndrome
Van der Sluis et al., 2015 [79]	New Function-Tailored Care Pathway for Elective TKR. Five elements: 1) Preoperative screening of physical functioning, 2) Postoperative monitoring of physical functioning, 3) Fast track tailored rehabilitation (twice-daily physiotherapy), 4) Communication with patient to improve self-efficacy, 5) Improvement of collaboration, communication and knowledge of HCP; versus usual care before implementation.	↓ Time to recovery of physical functioning*, ↓ Length of stay*
Zisberg et al., 2018 [80]	Walk FOR’ protocol to reduce barriers, to re-shape staff attitudes and knowledge, and to increase in-hospital mobility of older adults; versus usual care before implementation.	↑ Step count/day*, ↑ Patients achieved ≥900 steps/day*, ↑ HCP knowledge, behavior, and attitudes toward in-patient mobility*, ↑ Patient perceived staff (walking) encouragement*, ↑ Patient attitude (response to the phrase ‘I believe that increasing in-hospital mobility will improve my recovery’)*

*FFC-AC* Function Focused Care for Acute Care, *HCP* healthcare professionals, *PA* physical activity, *TKR* total knee replacement

^a^ = clinical intervention effect in favor of intervention group

Eleven studies reported improvements in clinical outcome variables, including an increase in patient step counts per day [49, 51, 56, 76, 80, 83]; more out-of-bed PA or less sedentary behavior [51, 53, 56, 77, 83] and improvements were seen in patients’ mobility level or physical functioning during a hospital stay [49, 51, 79].

## Discussion

In this systematic review of 48 studies, the identified facilitators and barriers of older patients’ PA behavior during hospitalization, from the perspective of older patients, caregivers, and HCPs, were multidimensional. The best evidence synthesis suggested that PA behavior is influenced by knowledge, awareness, and attitudes, including patients’ unwillingness or refusal to move, and by patients’ physical health status or medical treatment, including having symptoms or having lines. Furthermore, patients’ fears, such as falling and safety concerns, hampered mobilization. Social support positively stimulated patients’ PA,’ including encouragement from HCPs, and patients’ PA declined due to a lack of companion encouragement. In addition, caregivers could be more involved in patient mobilization. Moreover, HCPs expressed the need for consultation, clear roles, and team collaboration, as well as sufficient staff to motivate and help patients to increase PA. Furthermore, PA behavior was influenced by sufficient resources, including time and equipment, and an attractive physical environment, whereas lack of space hampered patients’ PA. Patient activities and awareness of PA protocols positively influenced mobilization, whereas zero-fall policies within hospitals restricted PA. Overall, identified facilitators and barriers spanned multiple levels of the social ecological framework, indicating patients’ PA behavior is complex and multifaceted.

In line with previous studies [27, 29, 91], the importance of awareness and knowledge about mobilization to improve patients’ PA behavior was recognized in the current review. However, when it comes to prioritizing poor health status or fear of falling, it seems that safety and zero-fall policies can sometimes take precedence over the known benefits of mobility. However, fatigue can actually be reduced by mobilization [24], and mobilization has the potential to reduce falls [25]. Therefore, since it might be possible for patients to mobilize, despite their health status, the focus on safety should not outweigh the benefits of PA [27], and PA policies should be adopted within the hospital culture, which might require a rethinking of the organization [7].

The current review showed that HCPs and caregivers can provide valuable support to increase patients’ mobility. Interestingly, the current review identified only two studies on the caregiver’s perspective, while in included studies [62, 67, 88] it was appointed several times by patients or HCPs to involve family by inpatient mobilization, e.g. by motivating and providing practical assistance. Despite the increased attention on PA in the last decade [92–94], there seems to be little focus on involving caregivers within the mobilization of older patients. In the intensive care setting, the concept of family involvement in inpatient mobilization is already gaining interest. Studies showed that family involvement has the potential to optimize patient outcomes, such as illness and recovery experience, as well as redirecting family psychological distress into an active participatory role, and supporting HCPs with the constraints of time and staffing [95–97]. Moreover, caregiver involvement may lead to better functional performance after discharge [98]. However, due to limited research on family participation in inpatient mobilization, the evidence on the effects of interventions that include family participation is low, and it is therefore difficult to draw strong conclusions.

In daily practice, clarifying roles and expectations of each other regarding mobilization appears to be complex among patients, caregivers, and HCPs. Older patients can take a passive role in their mobility and wait for encouragement from HCPs, while HCPs can see unwilling or unmotivated patients, leading to incorrect assumptions of each other. Unclear expectations of each other occur not only in older patients, but in all age groups [27, 91]. It is therefore important to tailor PA promotion, since patients may be waiting for instructions or assistance. Furthermore, despite interdisciplinary collaboration among staff, unclear responsibilities for mobility promotion tend to blur role clarity. It might be that mobility promotion is seen as a task of the physiotherapist and may not have been fully implemented by nurses [24] or other disciplines. Enabling PA should be a shared responsibility in which all professions have expertise that can promote patient mobility [99].

Modifiable factors at the institutional level, including sufficient resources, stimulating physical environment, and patient activities, were also reported in previous research and multidimensional interventions were suggested [27–29, 91, 100] which is in line with our results. Our review showed that multidimensional intervention studies, including an increase of resources, adding ambulation pathways, or monitoring physical functioning, might result in positive effects on patients’ step count per day [72, 80], or faster recovery of physical functioning [79]. Interestingly, in the identified PA interventions in current review, it seems that changing one element at institutional level might also act as a facilitator of PA and positively influences patient outcomes. For example, adding only extra ambulation sessions [49, 76, 78], or providing patient education [51]. Therefore, despite the fact that PA behavior is a complex phenomenon [39], starting with changing one element incorporated in daily care and tailored per ward, might also be a step forward to improve patients’ PA behavior.

This study had several strengths. First, both quantitative and qualitative data were included, as well as inclusion of the perspectives of patients, caregivers, and HCPs. To the best of our knowledge, this broad inclusion of study type and population has not been performed before and provides an enriched set of data. Second, many facilitators and barriers were reported by both study designs and across different geographical settings worldwide, improving the generalizability of our findings. Third, errors in the selection of studies, data extraction, quality appraisal, and data analysis were minimized by the involvement of at least two researchers. A limitation is that most all of our included studies took place in high income countries. Therefore, it is possible that important facilitators and barriers of low and middle income countries are underexposed, and future studies in these countries are needed. Secondly, in our review, palliative care patients were excluded. However, since many of them can benefit from mobilization, we suggest to include this population for future research. Furthermore, no meta-synthesis was performed. However, the best evidence synthesis was performed for the quantitative studies to weight the results and to provide insight into the level of evidence of our results. We are aware that these results show mostly insufficient or low evidence. It should be noted that conducting research in such a complex hospital environment and with a heterogeneous population makes it difficult to conduct strong methodological research studies with sufficient power. However, in our review, the evidence and reliability of the results of quantitative studies were enhanced by similar findings in qualitative studies. Furthermore, it could be argued that the best evidence synthesis results might have been influenced by the use of the MMAT, which in turn indicated a relatively large number of low methodological quality RCTs and non-randomized studies. Moreover, by using the MMAT, we were aware of the recommendation of the MMAT authors [35] to not calculating an overall total quality score since this may be a reason to exclude studies without specifying contents or criteria. In the current review, the use of a total score was not a limitation, since we included all studies despite low quality scores. Furthermore, a limitation of the MMAT might be the ‘can’t tell’ category, in which it is not clearly described whether a study has performed a certain criterion, or that it has not. The reader should be aware that this ambiguity might lead to underestimation or to overestimation of the overall quality results. Since it can be both ways, the overall quality scores might be more difficult to interpret. For future implications, high methodological quality studies should be conducted to increase the evidence for facilitators and barriers toward PA in older hospitalized patients.

In conclusion, the identified facilitators and barriers of PA behavior in older patients during hospitalization were multidimensional and spanned interpersonal, intrapersonal, and institutional levels. By including the perspectives of patients, caregivers, and HCPs, it was possible to provide a deeper insight into the complex interactions between those involved. However, it was found that facilitators and barriers were not extensively described from the perspective of caregivers, and future research on this perspective is warranted. Considering facilitators and barriers in a structured behavioral change framework might clarify potential strategies to enhance older patients’ PA behavior. We recommend that when enhancing PA in older hospitalized patients, attention should be paid to facilitators and barriers at the intrapersonal, interpersonal, and institutional levels, specifically targeting the health benefits of PA, patients’ health condition and willingness to move, healthcare team influences, resources, and hospital policies.

## Supplementary Information

**Additional file 1.** PRISMA 2020 Checklist for Systematic Reviews. Completed PRISMA 2020 for Systematic Reviews checklist for this systematic review.

**Additional file 2.** PRISMA 2020 Checklist for Abstract. Completed PRISMA 2020 for Abstracts checklist for this systematic review.

**Additional file 3.** Search strategy. Presentation of the used search terms with databases.

**Additional file 4.** Social ecological model with definitions. Presentation of levels of influence within the social ecological model.

**Additional file 5.** Characteristics of included studies. Table presenting the characteristics of included studies.

**Additional file 6.** Methodological quality. Table presenting detailed information of assessment of methodological quality of included studies.

**Additional file 7.** Overview of facilitators and barriers per (sub)theme. Table presenting an overview of all identified facilitators and barriers per (sub)theme and per perspective.

**Additional file 8.** Best-evidence synthesis classification. Table presenting an overview of the best-evidence synthesis classification for facilitators and/or barriers within quantitative studies.

## Data Availability

The datasets used and/or analyzed during the current study are available from the corresponding author on reasonable request.

## Abbreviations

ADL: Activities of daily living
HCPs: Healthcare professionals
MMAT: Mixed Methods Appraisal Tool
PA: Physical activity
RCTs: Randomized controlled trials
