# Supplementary material for: Facilitators and barriers to enhancing physical activity in older patients during acute hospital stay: a systematic review
Source: Int J Behav Nutr Phys Act. 2022 Jul 30;19:99. doi: 10.1186/s12966-022-01330-z (PMC9338465; doi:10.1186/s12966-022-01330-z)
Supplement: Supplementary file 3 — Additional file 3. Search strategy. Presentation of the used search terms with databases. [file 12966_2022_1330_MOESM3_ESM.docx]

**Additional file 3.** Search strategy.

May 29, 2021

**PubMed / MEDLINE (Ovid)**(("Hospitalization"[Mesh] OR "Inpatients"[Mesh] OR hospitalization[Title/Abstract] OR hospitalisation[Title/Abstract] OR “hospital stay”[Title/Abstract] OR hospitalized[Title/Abstract] OR hospitalised[Title/Abstract] OR “acute ward”[Title/Abstract] OR “hospital ward”[Title/Abstract] OR inpatient[Title/Abstract] OR inpatients[Title/Abstract] OR “hospital patient”[Title/Abstract] OR “hospital patients”[Title/Abstract]) AND ("Exercise"[Mesh] OR "Sedentary Behavior"[Mesh] OR "Walking"[Mesh] OR "Early Ambulation"[Mesh] OR "Motor Activity"[Mesh] OR "Locomotion"[Mesh] OR "Physical Therapy Modalities"[Mesh] OR "Self Care"[Mesh] OR "Mobility Limitation"[Mesh] OR “physical activity”[Title/Abstract] OR “physically active”[Title/Abstract] OR “physical active”[Title/Abstract] OR “physical inactivity”[Title/Abstract] OR “physically inactive”[Title/Abstract] OR “physical inactive”[Title/Abstract] OR exercise[Title/Abstract] OR lying[Title/Abstract] OR sitting[Title/Abstract] OR standing[Title/Abstract] OR walking[Title/Abstract] OR steps[Title/Abstract] OR stepping[Title/Abstract] OR mobilization[Title/Abstract] OR mobilisation[Title/Abstract] OR mobility[Title/Abstract] OR “physical function”[Title/Abstract] OR “physical functioning”[Title/Abstract] OR “physical mobility"[Title/Abstract] OR ambulation[Title/Abstract] OR "sedentary behavior"[Title/Abstract] OR "sedentary behaviour"[Title/Abstract] OR “motor activity”[Title/Abstract] OR locomotion[Title/Abstract] OR “self care”[Title/Abstract] OR selfcare[Title/Abstract] OR “self management”[Title/Abstract] OR selfmanagement[Title/Abstract] OR “immobility”[Title/Abstract] OR “physical effort”[Title/Abstract] OR “physical therapy”[Title/Abstract] OR “physiotherapy”[Title/Abstract]) AND ("Motivation"[Mesh] OR barrier[Title/Abstract] OR barriers[Title/Abstract] OR facilitator[Title/Abstract] OR facilitators[Title/Abstract] OR motivation[Title/Abstract] OR motivator[Title/Abstract] OR motivators[Title/Abstract] OR experience[Title/Abstract] OR experiences[Title/Abstract] OR perspective[Title/Abstract] OR perspectives[Title/Abstract] OR factor[Title/Abstract] OR factors[Title/Abstract]))) AND ((("Aged"[Mesh] OR "Aged, 80 and over"[Mesh] OR "Frail Elderly"[Mesh] OR "Geriatrics"[Mesh])) OR (elder[Title/Abstract] OR elderly[Title/Abstract] OR older[Title/Abstract] OR geriatric[Title/Abstract] OR geriatrics[Title/Abstract]))

**CINAHL (Ebsco)**

( ( MH ("Hospitalization" OR "Inpatients") ) OR TI ( hospitalization OR hospitalisation OR “hospital stay” OR hospitalized OR hospitalised OR “acute ward” OR “hospital ward” OR inpatient OR inpatients OR “hospital patient” OR “hospital patients” ) OR AB ( hospitalization OR hospitalisation OR “hospital stay” OR hospitalized OR hospitalised OR “acute ward” OR “hospital ward” OR inpatient OR inpatients OR “hospital patient” OR “hospital patients” ) ) AND ( ( MH ("Physical Activity" OR "Exercise" OR "Walking" OR "Physical Mobility" OR "Physical Therapy" OR "Motor Activity" OR "Locomotion" OR "Immobility" OR "Self Care") ) OR TI ( “physical activity” OR “physically active” OR “physical active” OR “physical inactivity” OR “physically inactive” OR “physical inactive” OR exercise OR lying OR sitting OR standing OR walking OR steps OR stepping OR mobilization OR mobilisation OR mobility OR “physical function” OR “physical functioning” OR “physical mobility" OR ambulation OR "sedentary behavior" OR "sedentary behaviour" OR “motor activity” OR locomotion OR “self care” OR selfcare OR “self management” OR selfmanagement OR “immobility” OR “physical effort” OR “physical therapy” OR “physiotherapy” ) OR AB ( “physical activity” OR “physically active” OR “physical active” OR “physical inactivity” OR “physically inactive” OR “physical inactive” OR exercise OR lying OR sitting OR standing OR walking OR steps OR stepping OR mobilization OR mobilisation OR mobility OR “physical function” OR “physical functioning” OR “physical mobility" OR ambulation OR "sedentary behavior" OR "sedentary behaviour" OR “motor activity” OR locomotion OR “self care” OR selfcare OR “self management” OR selfmanagement OR “immobility” OR “physical effort” OR “physical therapy” OR “physiotherapy” ) ) AND ( ( MH "Motivation" OR TI ( barrier OR barriers OR facilitator OR facilitators OR motivation OR motivator OR motivators OR experience OR experiences OR perspective OR perspectives OR factor OR factors ) OR AB ( barrier OR barriers OR facilitator OR facilitators OR motivation OR motivator OR motivators OR experience OR experiences OR perspective OR perspectives OR factor OR factors ) ) AND ( ( MH ("Aged" OR "Aged, 80 and Over" OR "Frail Elderly" OR "Aged, Hospitalized") ) OR TI ( elder OR elderly OR older OR geriatric OR geriatrics ) OR AB ( elder OR elderly OR older OR geriatric OR geriatrics )

**PsycINFO (Ebsco)**

( ( DE ( "Hospitalization" OR "Patients" ) ) OR TI ( hospitalization OR hospitalisation OR “hospital stay” OR hospitalized OR hospitalised OR “acute ward” OR “hospital ward” OR inpatient OR inpatients OR “hospital patient” OR “hospital patients” ) OR AB ( hospitalization OR hospitalisation OR “hospital stay” OR hospitalized OR hospitalised OR “acute ward” OR “hospital ward” OR inpatient OR inpatients OR “hospital patient” OR “hospital patients” ) ) AND ( ( DE ( "Physical Activity" OR "Exercise" OR "Walking" OR "Physical Mobility" OR "Locomotion” OR "Physical Therapy") ) OR TI ( “physical activity” OR “physically active” OR “physical active” OR “physical inactivity” OR “physically inactive” OR “physical inactive” OR exercise OR lying OR sitting OR standing OR walking OR steps OR stepping OR mobilization OR mobilisation OR mobility OR “physical function” OR “physical functioning” OR “physical mobility" OR ambulation OR "sedentary behavior" OR "sedentary behaviour" OR “motor activity” OR locomotion OR “self care” OR selfcare OR “self management” OR selfmanagement OR “immobility” OR “physical effort” OR “physical therapy” OR “physiotherapy” ) OR AB ( “physical activity” OR “physically active” OR “physical active” OR “physical inactivity” OR “physically inactive” OR “physical inactive” OR exercise OR lying OR sitting OR standing OR walking OR steps OR stepping OR mobilization OR mobilisation OR mobility OR “physical function” OR “physical functioning” OR “physical mobility" OR ambulation OR "sedentary behavior" OR "sedentary behaviour" OR “motor activity” OR locomotion OR “self care” OR selfcare OR “self management” OR selfmanagement OR “immobility” OR “physical effort” OR “physical therapy” OR “physiotherapy” ) ) AND ( ( DE "Motivation" OR TI ( barrier OR barriers OR facilitator OR facilitators OR motivation OR motivator OR motivators OR experience OR experiences OR perspective OR perspectives OR factor OR factors ) OR AB ( barrier OR barriers OR facilitator OR facilitators OR motivation OR motivator OR motivators OR experience OR experiences OR perspective OR perspectives OR factor OR factors ) ) AND ( (TI ( elder OR elderly OR older OR geriatric OR geriatrics ) OR AB ( elder OR elderly OR older OR geriatric OR geriatrics ) )

**Embase (Ovid)**

('hospitalization'/exp OR 'hospital patient'/exp OR hospitalization:ti,ab OR hospitalisation:ti,ab OR 'hospital stay':ti,ab OR hospitalized:ti,ab OR hospitalised:ti,ab OR 'acute ward':ti,ab OR 'hospital ward':ti,ab OR ‘inpatient’:ti,ab OR ‘inpatients’:ti,ab OR 'hospital patient':ti,ab OR 'hospital patients':ti,ab) AND ('physical activity'/exp OR 'exercise'/exp OR 'walking'/exp OR 'mobilization'/exp OR 'physical mobility'/exp OR 'motor activity'/exp OR 'locomotion'/exp OR 'self care'/exp OR 'immobility'/exp OR 'physiotherapy'/exp OR 'physical activity':ti,ab OR 'physically active':ti,ab OR 'physical active':ti,ab OR 'physical inactivity':ti,ab OR 'physically inactive':ti,ab OR 'physical inactive':ti,ab OR exercise:ti,ab OR lying:ti,ab OR sitting:ti,ab OR standing:ti,ab OR walking:ti,ab OR steps:ti,ab OR stepping:ti,ab OR mobilization:ti,ab OR mobilisation:ti,ab OR mobility:ti,ab OR 'physical function':ti,ab OR 'physical functioning':ti,ab OR ‘physical mobility’:ti,ab OR ambulation:ti,ab OR ‘sedentary behavior’:ti,ab OR ‘sedentary behaviour’:ti,ab OR ‘motor activity’:ti,ab OR locomotion:ti,ab OR ‘self care’:ti,ab OR selfcare:ti,ab OR ‘self management’:ti,ab OR selfmanagement:ti,ab OR ‘immobility’:ti,ab OR ‘physical effort’:ti,ab OR ‘physical therapy’:ti,ab OR ‘physiotherapy’:ti,ab) AND ('motivation'/exp OR barrier:ti,ab OR barriers:ti,ab OR facilitator:ti,ab OR facilitators:ti,ab OR motivation:ti,ab OR motivator:ti,ab OR motivators:ti,ab OR experience:ti,ab OR experiences:ti,ab OR perspective:ti,ab OR perspectives:ti,ab OR factor:ti,ab OR factors:ti,ab) AND ('aged'/exp OR 'very elderly'/exp OR 'frail elderly'/exp OR 'geriatric patient'/exp OR elder:ti,ab OR elderly:ti,ab OR older:ti,ab OR geriatric:ab,ti OR geriatrics:ab,ti)

**Web of Science (Ebsco)**

TS=(hospitalization OR hospitalisation OR “hospital stay” OR hospitalized OR hospitalised OR “acute ward” OR “hospital ward” OR inpatient OR inpatients OR “hospital patient” OR “hospital patients”) AND TS=(“physical activity” OR “physically active” OR “physical active” OR “physical inactivity” OR “physically inactive” OR “physical inactive” OR exercise OR lying OR sitting OR standing OR walking OR steps OR stepping OR mobilization OR mobilisation OR mobility OR “physical function” OR “physical functioning” OR “physical mobility" OR ambulation OR "sedentary behavior" OR "sedentary behaviour" OR “motor activity” OR locomotion OR “self care” OR selfcare OR “self management” OR selfmanagement OR “immobility” OR “physical effort” OR “physical therapy” OR “physiotherapy”) AND TS=(barrier OR barriers OR facilitator OR facilitators OR motivation OR motivator OR motivators OR experience OR experiences OR perspective OR perspectives OR factor OR factors) AND TS=(elder OR elderly OR older OR geriatric OR geriatrics)
