# Supplementary material for: Facilitators and barriers to enhancing physical activity in older patients during acute hospital stay: a systematic review
Source: Int J Behav Nutr Phys Act. 2022 Jul 30;19:99. doi: 10.1186/s12966-022-01330-z (PMC9338465; doi:10.1186/s12966-022-01330-z)
Supplement: Supplementary file 4 — Additional file 4. Social ecological model with definitions. Presentation of levels of influence within the social ecological model. [file 12966_2022_1330_MOESM4_ESM.docx]

**Additional file 4.** Social ecological model^a^ with definitions.

| **Concept** |  | **Definition** |
| --- | --- | --- |
| Intrapersonal level |  | Individual characteristics that influence behavior, such as knowledge, attitudes, beliefs, and personality traits |
| Interpersonal level |  | Interpersonal processes and primary groups, including family, friends, and peers that provide social identity, support, and role definition |
| Institutional level | Institutional factors | Rules, regulations, policies, and informal structures, which may constrain or promote recommended behaviors |
|  | Community factors | Social networks and norms, or standards, which exist as formal or informal among individuals, groups, and organizations |
|  | Public policy factors | Local, state, and federal policies and laws that regulate or support healthy actions and practices for disease prevention, early detection, control, and management |
| ^a^ Adapted from McLeroy et al. (1988) | |  |
