# Supplementary material for: Facilitators and barriers to enhancing physical activity in older patients during acute hospital stay: a systematic review
Source: Int J Behav Nutr Phys Act. 2022 Jul 30;19:99. doi: 10.1186/s12966-022-01330-z (PMC9338465; doi:10.1186/s12966-022-01330-z)
Supplement: Supplementary file 5 — Additional file 5. Characteristics of included studies. Table presenting the characteristics of included studies. [file 12966_2022_1330_MOESM5_ESM.docx]

**Additional file 5.** Characteristics of the included studies (n=48).

| **Author, year** | **Study country** | **Setting** | **Aim or research question of study** | **Study design** | **Study population^a^** | **Quality appraisal MMAT score^b^** |
| --- | --- | --- | --- | --- | --- | --- |
| Agostini et al., 2014 | UK | Thoracic surgical ward in a tertiary regional thoracic center. | To determine how physically active patients were immediately following major thoracic surgery, and to identify any specific factors contributing to possible limitation. | QUAN – prospective observational study. | 99 patients, 53 F, 67.0 ± 10 yrs. | 3 |
| Andreasen et al., 2018 | Denmark | Medical ward specializing in infectious diseases in a university hospital. | To identify positive and negative factors affecting the adherence of patients and staff to an integrated physical activity and nutritional intervention on a medical ward. | QUAL – semi structured focus groups and individual interviews. | 7 patients, 5 F, 69.5 ± 6.5 yrs.  5 nursing staff, 5 F, 34.6 ± 8.7 yrs. | 5 |
| Babine et al., 2019 | USA | International hospitals on the official HELP site registrant list. | To describe characteristics of the HELP sites worldwide, number of patient enrollments, type of mobilization, and fall and fall injury rates and to identify barriers to mobilization. | QUAN – observational study. | 28 contact persons of hospitals settings working with HELP, n/a, n/a. | 0 |
| Belala et al., 2019 | Germany | Geriatric acute care ward of a German hospital. | To describe contextual factors and circumstances via direct observation in order to understand cognitively impaired inpatients’ activity behavior during acute hospitalization. | MM – observational design and semi structured individual interviews. | Observational  20 patients, 12 F, 84.0 ± 6.8 yrs.   Interviews  5 HCPs, 5 F, 32.5 ± 5.6 yrs.  - including one employee of each profession (physician, OT, PT, nurse, service staff). | 1 |
| Boltz et al., 2010 | USA | Not specified. | The intent was to acquire insight into the factors influencing physical function during hospitalization, as perceived by older adult consumers, by developing contextual descriptions of these areas. | QUAL – Retrospective, semi structured focus group interviews. | 24 patients, 14 F, 72-94 yrs. | 5 |
| Boltz et al., 2011 | USA | Medical, surgical, emergency, critical care, and specialty unit in a suburban community hospital and a urban teaching hospital. | To discover the organizational barriers and facilitators, as perceived by nursing staff, which influence physical function in hospitalized older adults. | QUAL – semi structured focus group interviews. | 55 HCPs, n/a, n/a.  - including 43 nurses, 12 patient care associates. | 5 |
| Brown et al., 2007 | USA | Medical wards in an university hospital. | 1) To identify and contextualize perceived barriers to mobility during hospitalization from the perspectives of older patients, their primary nurses, and their resident physicians; 2) To compare and contrast the perceived barriers among these three groups; and 3) To compare perceived barriers to mobility with our conceptual model. | QUAL – semi structured individual interviews. | 10 patients, 7 F, 84 ± 6.0 yrs.  10 nurses, 10 F, 34.9 ± 9.9 yrs. 9 physicians, 4 F, 29.1 ± 2.2 yrs. | 5 |
| Chan et al., 2019 | Singapore | Various general wards in a large 1300 bedded acute hospital. | To explore nurses' perceptions of older patients' physical activity in an acute hospital setting in Singapore. | QUAL – semi structured focus group interviews. | 30 nurses, 28 F, 28.27 ± 6.0 yrs. | 5 |
| Chase et al., 2018 | USA | Various wards in six hospitals. | To investigate a broad range of factors associated with a decline in mobility, defined by the ambulation component of basic ADLs, among hospitalized older adults. | QUAN – secondary data analysis of observational data. | 959 patients, 537 F, 78.14 ± 8.3 yrs. | 4 |
| Chua et al., 2017 | Australia | 19 high-volume hip or knee arthroplasty providing hospitals. | To determine 1) the proportions of patients that first mobilized on post-operative day 0 (POD 0) and 2) factors associated with earlier time to mobilization. | QUAN – prospective observational study. | 1807 patients - 818 THA, 431 F, 68.3 ± 8.7 yrs.  - 989 TKA, 557 F, 66.9 ± 9.7 yrs. | 5 |
| De Klein et al., 2019 | the Netherlands | Geriatrics and gastroenterology wards in a large university hospital. | To give an overview of factors that influence physical activity of patients by exploring the perspectives of both patients and health-care professionals regarding physical activity during hospital stay. | QUAL – semi structured individual interviews. | 8 patients, 2 F, mean age 69.8, range 49-87 yrs.  9 HCPs, 6 F, mean age 31.0, range 24-60 yrs. | 5 |
| Dermody et al., 2017 | USA | Variety of non-intensive care units in two community-based hospitals. | 1) To examine nurses' perceptions of barriers to promoting physical activity in hospitalized older adults and 2) to determine differences in perceptions of barriers between nurses based on nurse experience and hospital unit. | QUAN – descriptive correlation study. | 85 nurses, 73 F, n/a. | 1 |
| Dermody et al., 2018 | USA | Variety of non-intensive care units in two community-based hospitals. | 1) To examine the association of nurses' knowledge, attitude, and external barriers on the promotion of mobility in hospitalized older patients in non-intensive care units; and 2) To examine the relationship between patient impairment of mobility, use of mobility assistive devices at home, being classified as risk for falls and nurse-promoted mobility. | QUAN – secondary data analysis with a cross-sectional descriptive correlation design. | 77 patients, 36 F, 78.4 ± 7.9 yrs. 61 nurses, 53 F, 40.5 ± 11.6 yrs. | 3 |
| Doherty-King et al., 2011 | USA | Adult medical or surgical units in two urban teaching hospitals. | To explore how nurses make decisions about ambulating hospitalized older adults. | QUAL – in depth individual interviews. | 25 registered nurses, n/a, n/a. | 5 |
| Evensen et al., 2017 | Norway | Geriatric ward in a university hospital. | 1) To describe a population of hospitalized geriatric patients and their level of physical activity measured by activity monitors; 2) To explore if physical function, age, diagnosis of cognitive impairment, function of personal Activities of Daily Living (p-ADL) and comorbidity are associated with physical activity during hospitalization. | QUAN – observational study. | 38 patients, 26 F, 82.9 ± 6.3 yrs. | 3 |
| Feenstra et al., 2021 | the Netherlands | Geriatric ward in a Dutch hospital | What is the impact of the applied changes to the spatial environment of the nursing ward on the activation of patients in the hospital? | MM – observational design, questionnaires and focus group interviews. | Observational 23 patients - before intervention group: 8, n/a, n/a. - after intervention group: 15, n/a, n/a.   Interviews n/a HCPs, n/a, n/a.  10 patients, n/a, n/a | 0 |
| Fisher et al., 2011 | USA | Acute Care for Elders (ACE) unit in an university teaching hospital. | To identify subgroups of patients with shared clinical profiles who differed with respect to mean daily ambulation. | QUAN – observational cohort study. | 198 patients, 108 F, 76.9 ± 7.8 yrs. | 2 |
| Haines et al., 2013 | Australia | Surgical wards in a tertiary teaching hospital. | 1) To record the incidence of pulmonary complications and test whether clinical variables were risk factors for the diagnosis of postoperative pulmonary complications; 2) To document the nature and timing of the physiotherapy intervention provided to the POST abdominal surgery cohort, and 3) identify barriers that delayed or prevented mobilization of patients in the early (day 1 to 7) postoperative period. | QUAN – prospective observational cohort study. | 72 patients, 27 F, 66.1 ± 12.4 yrs. | 5 |
| Hamilton et al., 2019 | USA | Various wards in a 1440 bedded tertiary care center. | To assess the feasibility and effectiveness of dedicated mobility technician-assisted ambulation in older inpatients. | QUAN – randomized controlled study. | 102 patients - 50 intervention group, 27 F, 75.6 ± 9.6 yrs. - 52 control group, 36 F, 76.8 ± 8.6 yrs. | 1 |
| Hartley et al., 2020 | UK | Tertiary university hospital. | To investigate clinical predictors of inhospital activity during the first 24 h of hospital admission in older adults in the United Kingdom (UK) using the innovative method of best-subset analysis. | QUAN – secondary data analysis of a prospective repeated measures cohort study. | 62 patients, 26 F, median age 85.0, IQR 80.2-87.0. | 3 |
| Haslam-Larmer et al., 2021 | Canada | Large tertiary care center. | To 1) describe early mobility activities on one post-operative unit with a history of recommendation implementation, and 2) identify factors influencing participation in early mobility activities after hip fracture surgery. | MM – descriptive embedded case study design. Observational design and semi structured individual interviews. | 19 patients in total, 14 F, 83.2 ± 10.5 yrs.   Observational 18 patients, n/a, n/a.   Interviews 18 patients, n/a, n/a.  10 HCPs, 6 F, n/a - including 2 physiotherapists, 2 occupational therapists, 1 therapy assistant, 5 registered nurses. | 2 |
| Ishikawa et al., 2020 | Japan | University Hospital. | To 1) examine the number of days required until ambulation in HF patients who were obliged to have bed rest on admission, and investigated whether delayed ambulation was associated with adverse cardiovascular events, and 2) identify factors related to delayed ambulation in those clinical settings. | QUAN – retrospective observational study. | 101 patients, 71 F, 66.0 ± 17 yrs. | 3 |
| Kavanagh et al., 2019 | Australia | Medical and surgical wards in five international and four Victorian acute hospitals. | To determine perceived barriers and facilitators to adopting and sustaining functional maintenance initiatives for acutely hospitalized older adults. | QUAL – semi structured focus group and individual interviews, and initiative observations. | 27 HCPs - 22 international, 14 F, mean age 53.0, range 24-70 yrs.  - 5 local, 4 F, mean age 32.0, range 21-51 yrs. | 5 |
| King et al., 2016 | USA | Adult general medical unit at a 648 bedded tertiary academic teaching hospital. | To develop a system-based intervention including five components that target barriers to nurse-initiated patient ambulation. | MM – observational design and semi-structured focus group and individual interviews. | Observational  n/a patients, n/a, n/a.  Interviews  16 nurses - 11 registered nurses, n/a, n/a. - 5 certified nurse assistants, n/a, n/a. | 1 |
| King et al., 2021 | USA | Not specified. | To understand older adults’ perceptions of and experiences with ambulation during a hospital admission. | QUAL – semi structured focus groups interviews. | 11 patients, n/a, n/a. | 3 |
| Kirk et al., 2019 | Denmark | Various medical departments in three public hospitals. | To explore how social contextual circumstances affect the mobility of older medical patients in medical departments. | QUAL – ethnographic study. Observations with go-along interviews. | 79 HCPs - 12 physiotherapists, 11 F, n/a.  - 33 registered nurses, 33 F, n/a.  - 16 nursing assistants, 16 F, n/a.  - 18 physicians, n/a, n/a. | 5 |
| McCullagh et al., 2020 | Ireland | All wards in a 350 bedded general teaching hospital. | To 1) identify potential factors that may explain inpatients' walking activity, and 2) measure the effect of time-invariant and time-varying factors on walking (average daily step count) during hospitalization. | QUAN – secondary data analysis of observational data. | 154 patients, 73 F, 77.5 ± 7.4 yrs. | 5 |
| Moore et al., 2014 | Canada | Inpatient units in 14 hospitals. | To develop a mapping guide that links identified barriers and intervention activities to behavior change theory. | QUAL – focus group interviews. | 261 HCPs, n/a, n/a. | 3 |
| Moreno et al., 2019 | Brazil | Respiratory and clinical medicine wards in an university hospital. | 1) To evaluate the impact of an orientation program for older hospital inpatients about the importance of staying physically active during hospitalization; 2) To identify the main barriers to staying physically active during hospitalization. | QUAN – randomized controlled trial. | 68 patients - 33 experimental group, 17 F, 69.0 ± 7.0 yrs.  - 35 control group, 11 F, 69.0 ± 7.0 yrs. | 5 |
| Mudge et al., 2015 | Australia | General medical ward in a 900 bedded teaching hospital. | This report describes Eat Walk Engage, a collaborative care model on a general medical ward in Brisbane, Australia. | MM – descriptive implementation study and interviews. | Descriptive design n/a patients, n/a, n/a.   Interviews 11 HCPs, n/a, n/a.  - 3 nurses, 7 allied health professionals, 1 physician. | 0 |
| O'Hare et al., 2017 | Ireland | Acute teaching hospital. | To explore how frail older inpatients engaged in, perceived and were influenced by a pilot augmented prescribed exercise program (APEP) conducted by McCullagh et al., (2014). | QUAL – semi structured individual interviews. | 13 patients, 8 F, mean age 75.6, range 65-85 yrs. | 4 |
| Pavon et al., 2021 | USA | General medicine ward at an academic tertiary medical center and academic community hospital. | To report on the perspectives of both patients and hospital providers, including physical and occupational therapists who are integral to mobility safety training and evaluation in hospitals, regarding: (1) factors that influence hospital mobility in older adults, and (2) how these factors can inform the design or implementation of hospital walking or mobility programs. | QUAL – semi structured focus groups and individual interviews. | 19 patients, 13 F, 77 ± 7.6 yrs.  48 HCPs, 40 F, 37.0 ± 10.1 yrs.  - including hospitalists, nurses, and physical and occupational therapists. | 5 |
| Pedersen et al., 2020 | Denmark | Medical departments at two hospitals. | To classify the most common barriers and facilitators to physicians’ promotion of mobility in older medical patients as part of an intervention to promote mobility using the Theoretical Domains Framework. | QUAL – semi structured individual interviews. | 12 physicians, 7 F, n/a. | 5 |
| Porserud et al., 2019 | Sweden | Surgical wards in an university hospital. | To evaluate the Activity board as a standardized method to enhance mobilization and postoperative recovery after abdominal surgery due to cancer. | QUAN – non randomized controlled trial. | 133 patients - 67 experimental group, 32 F, 69.3 ± 11.4 yrs.  - 66 control group, 34 F, 67.0 ± 13.1 yrs. | 4 |
| Resnick et al., 2015 | USA | Trauma units in two trauma teaching hospitals. | To describe 1) the initial implementation process for FFC-AC; 2) the status of the first 25 participants; 3) To explore challenges to optimizing physical activity among these individuals. | QUAN – descriptive study. | 25 patients, 18 F, 79.6 ± 8.8 yrs.  114 nurses - 62 intervention group (FFC-AC), n/a, n/a.  - 51 education only group (FFC-ED), n/a, n/a. | 2 |
| S. Lim et al., 2020 | UK | Acute medical wards in one UK hospital. | 1) To determine the feasibility and acceptability of a volunteer-led mobility intervention in an acute care setting; 2) To explore the impact of the intervention on patient outcomes. | MM – pre-post mixed-methods study. Observational design, focus group and individual interviews. | Observational  100 patients - 50 baseline group, n/a, 87.2 ± 4.6 yrs. - 50 intervention group, n/a, 86.2 ± 5.1 yrs.  Interviews  25 participants - 6 patients, 3 F, 82-94 yrs. - 6 nurses, 4 F, n/a. - 7 therapists, 7 F, n/a.  - 6 volunteers, 4 F, 17-62 yrs. | 2 |
| S.H. Lim et al., 2020 | Singapore | General medical ward in a tertiary public hospital. | To explore patients’, their family carers’, and nurses’ perceptions of promotion of mobility among hospitalized older adults. | QUAL – semi structured individual interviews. | 14 patients, 12 F, 69-87 yrs.  6 caregivers, 4 F, 39-61 yrs. 10 nurses, n/a, n/a. | 5 |
| Said et al., 2021 | Australia | Orthopedic unit in a single Australian tertiary health service. | To explore 1) the proportion of patients’ postsurgical fixation of hip fracture who mobilize away from the bedside in the first 48 hours post surgery, 2) factors associated with mobilization within 48 hours post surgery, and 3) barriers to mobilization post surgery, as identified by the treating physical therapist. | QUAN – prospective observational study. | 100 patients, 66 F, 82 ± 9 yrs.  n/a physical therapist, n/a, n/a. | 4 |
| Scheerman et al., 2020 | the Netherlands | Various wards in an academic teaching hospital. | To investigate how nurses perceive tasks and responsibilities in physical activity promotion of hospitalized older patients and which factors are of influence. | MM – sequential explanatory design. Questionnaire survey and semi structured individual interviews. | Survey 108 nurses - 13 nurse students, 11 F, median age 25, IQR 23.0-29.0 yrs.  - 85 nurses, 73 F, median age 32, IQR 25.0-51.0 yrs.  - 10 nurse supervisors, 10 F, median age 49, IQR 42.8-54.5 yrs.   Interviews 51 nurses, 44 F, median age 31, IQR 26.0-45.0 yrs. | 2 |
| Scheerman et al., 2021 | the Netherlands | Various wards in an academic teaching hospital. | What physical, motivational and environmental barriers and enablers of physical activity promotion are perceived by older patients during hospitalization and if perceived barriers and enablers differ in physically dependent and independent older patients? | MM – questionnaire survey and semi structured individual interviews. | 49 patients, 25 F, median age 77, IQR 75.0-83.0 yrs. | 2 |
| Shannon et al., 2019 | Australia | Neurological wards in two acute hospitals. | To explore how the physical environment in a new ward might be associated with neurological patient physical and social activities in comparison with an older ward environment. | QUAN – prospective observational study using a 'before and after' methodology. | 37 patients - 17 old ward, 7 F, 69-85 yrs.  - 20 new ward, 8 F, 57-81 yrs. | 1 |
| So et al., 2012 | USA | General medical and surgical wards in a 282 bedded public teaching hospital. | 1) To describe the attitudes toward and expectations of hospitalized older adults regarding exercise in the hospital; 2) To explore motivators and barriers to exercise in the hospital; and 3) To characterize the role of physicians and nurses in promoting in-hospital exercise. | QUAL – semi structured individual interviews. | 28 patients, 13 F, mean age 77.7, range 65-103 yrs. | 5 |
| Stefánsdóttir et al., 2021 | Denmark | Endocrinology and general medicine departments at two hospitals. | To explores older medical patients’ experiences with participating in the multi-component mobility intervention of the WALK Copenhagen (WALK-Cph) project and in-hospital mobility more generally. | QUAL – semi structured individual interviews. | 20 patients, 11 F, 77.0 ± 8.0 yrs. | 5 |
| Sun et al., 2019 | USA | Cancer center hospital. | To explore barriers and facilitators of adherence to a perioperative physical activity intervention for older adults with lung and gastrointestinal (GI) cancers and their family caregivers (FCGs), in order to gain better insight into factors that may predict intervention engagement. | QUAL – analysis of PT/OT notes. | 34 patients, 14 F - 16 GI surgery, median age 74 yrs.  - 18 lung surgery, median age 68 yrs. 34 caregivers (cg), 20 F - 16 GI surgery cg, median age 71 yrs.  - 18 lung surgery cg, median age 67 yrs. | 3 |
| Tousignant-Laflamme et al., 2015 | Canada | Emergency department and family medicine ward. | 1) What are the barriers and facilitators to the implementation of PT services in the ED? and 2) What is the potential clinical value of adding PT services to the ED, in collaboration with nursing staff, as a mean of preventing IS in older persons 65 years and over with at least one clinical sign of impaired mobility? | MM – observational design and interviews. | Observational 20 patients  - 9 with treatment, n/a, n/a.  - 11 with assessment only, n/a, n/a.   Interviews 7 HCP, n/a, n/a. | 1 |
| Van Der Sluis et al., 2015 | the Netherlands | Orthopedic ward in a 320 bedded regional hospital. | To study whether a function-tailored care pathway compared to the usual care situation before its introduction was able to reduce the time needed to achieve functional independence during hospital stay and length of in-hospital stay. | MM – observational cohort study design and semi structured individual interviews. | Observational 235 patients, 170 F, 70.8 yrs.  -127 before group, 94 F, 71.1 yrs.  - 108 after group, 76 F, 70.4 yrs.   Interviews 8 HCP, 8 F, n/a. | 1 |
| Zisberg et al., 2016 | Israel | Acute care units in two medical centers. | To test the relationship of satisfaction with hospital environment, sleep-medication consumption, and in-hospital caloric intake to in-hospital mobility levels. | QUAN – prospective observational study. | 769 patients, 394 F, 78.6 ± 5.8 yrs. | 2 |
| Zisberg et al., 2018 | Israel | Internal medicine units in an academic medical center. | To demonstrate the process of adapting a human factors framework, the Systems Engineering Initiative for Patient Safety (SEIPS 2.0), as a guided model to articulate a site-specific, culturally based intervention to improve in-hospital mobility in older adults. | MM – observational design, focus groups and individual interviews. | Observational 203 patients, n/a, n/a. 116 HCP, n/a, n/a.   Interviews 11 key persons in leadership positions, n/a, n/a.  n/a HCP. | 1 |

Abbreviations: F, female; FFC-AC, Function Focused Care for Acute Care; GI, gastrointestinal; HCP, healthcare professionals; MM, mixed-methods study design; n/a, not applicable; QUAL, qualitative research; QUAN, quantitative research; THA, total hip arthroplasty; TKA, total knee arthroplasty; yrs, years;

^a^ Study population described as: number of participants, number of females, age (mean ± SD, range).

^b^ Mixed-methods appraisal tool, version 2018. Scores varied from 0 – 5.
