# Supplementary material for: Facilitators and barriers to enhancing physical activity in older patients during acute hospital stay: a systematic review
Source: Int J Behav Nutr Phys Act. 2022 Jul 30;19:99. doi: 10.1186/s12966-022-01330-z (PMC9338465; doi:10.1186/s12966-022-01330-z)
Supplement: Supplementary file 6 — Additional file 6. Methodological quality. Table presenting detailed information of assessment of methodological quality of included studies. [file 12966_2022_1330_MOESM6_ESM.docx]

**Additional file 6.** Assessment of methodological quality of included studies (n=48) using Mixed Methods Appraisal Tool, version 2018.

| **First author, year** | **Criteria from the Mixed Methods Appraisal Tool, version 2018** | | | | | | | | | | | | | | | | | | | | | | | | | | | | | **Overall quality score, 0-5** |
| --- | --- | --- | --- | --- | --- | --- | --- | --- | --- | --- | --- | --- | --- | --- | --- | --- | --- | --- | --- | --- | --- | --- | --- | --- | --- | --- | --- | --- | --- | --- |
|  | **Qualitative studies** | | | | |  | **Quantitative studies** | | | | | | | | | | | | | | | | |  | **Mixed-methods studies** | | | | |  |
|  |  | | | | |  | **RCT** | | | | |  | **Non-randomized** | | | | |  | **Descriptive** | | | | |  |  | | | | |  |
|  | 1.1 | 1.2 | 1.3 | 1.4 | 1.5 |  | 2.1 | 2.2 | 2.3 | 2.4 | 2.5 |  | 3.1 | 3.2 | 3.3 | 3.4 | 3.5 |  | 4.1 | 4.2 | 4.3 | 4.4 | 4.5 |  | 5.1 | 5.2 | 5.3 | 5.4 | 5.5 |  |
| Agostini, 2014 |  |  |  |  |  |  |  |  |  |  |  |  | Y | Y | N | Y | ? |  |  |  |  |  |  |  |  |  |  |  |  | 3 |
| Andreasen, 2018 | Y | Y | Y | Y | Y |  |  |  |  |  |  |  |  |  |  |  |  |  |  |  |  |  |  |  |  |  |  |  |  | 5 |
| Babine, 2019 |  |  |  |  |  |  |  |  |  |  |  |  |  |  |  |  |  |  | N | N | N | N | ? |  |  |  |  |  |  | 0 |
| Belala, 2019 | N | N | ? | Y | ? |  |  |  |  |  |  |  | N | Y | Y | N | ? |  |  |  |  |  |  |  | Y | Y | Y | ? | N | 1 |
| Boltz, 2010 | Y | Y | Y | Y | Y |  |  |  |  |  |  |  |  |  |  |  |  |  |  |  |  |  |  |  |  |  |  |  |  | 5 |
| Boltz, 2011 | Y | Y | Y | Y | Y |  |  |  |  |  |  |  |  |  |  |  |  |  |  |  |  |  |  |  |  |  |  |  |  | 5 |
| Brown, 2007 | Y | Y | Y | Y | Y |  |  |  |  |  |  |  |  |  |  |  |  |  |  |  |  |  |  |  |  |  |  |  |  | 5 |
| Chan, 2019 | Y | Y | Y | Y | Y |  |  |  |  |  |  |  |  |  |  |  |  |  |  |  |  |  |  |  |  |  |  |  |  | 5 |
| Chase, 2018 |  |  |  |  |  |  |  |  |  |  |  |  | Y | ? | Y | Y | Y |  |  |  |  |  |  |  |  |  |  |  |  | 4 |
| Chua, 2017 |  |  |  |  |  |  |  |  |  |  |  |  | Y | Y | Y | Y | Y |  |  |  |  |  |  |  |  |  |  |  |  | 5 |
| De Klein, 2019 | Y | Y | Y | Y | Y |  |  |  |  |  |  |  |  |  |  |  |  |  |  |  |  |  |  |  |  |  |  |  |  | 5 |
| Dermody, 2017 |  |  |  |  |  |  |  |  |  |  |  |  | N | N | Y | N | N |  |  |  |  |  |  |  |  |  |  |  |  | 1 |
| Dermody, 2018 |  |  |  |  |  |  |  |  |  |  |  |  | N | Y | Y | ? | Y |  |  |  |  |  |  |  |  |  |  |  |  | 3 |
| Doherty-King, 2011 | Y | Y | Y | Y | Y |  |  |  |  |  |  |  |  |  |  |  |  |  |  |  |  |  |  |  |  |  |  |  |  | 5 |
| Evensen, 2017 |  |  |  |  |  |  |  |  |  |  |  |  | ? | Y | N | Y | Y |  |  |  |  |  |  |  |  |  |  |  |  | 3 |
| Feenstra et al., 2021 | Y | N | ? | ? | ? |  |  |  |  |  |  |  | ? | ? | ? | ? | ? |  |  |  |  |  |  |  | Y | N | N | N | N | 0 |
| Fisher, 2012 |  |  |  |  |  |  |  |  |  |  |  |  | Y | ? | ? | N | Y |  |  |  |  |  |  |  |  |  |  |  |  | 2 |
| Haines, 2013 |  |  |  |  |  |  |  |  |  |  |  |  | Y | Y | Y | Y | Y |  |  |  |  |  |  |  |  |  |  |  |  | 5 |
| Hamilton, 2019 |  |  |  |  |  |  | ? | N | Y | N | N |  |  |  |  |  |  |  |  |  |  |  |  |  |  |  |  |  |  | 1 |
| Hartley et al., 2020 |  |  |  |  |  |  |  |  |  |  |  |  | N | Y | Y | ? | Y |  |  |  |  |  |  |  |  |  |  |  |  | 3 |
| Haslam-Larmer et al., 2021 | Y | Y | Y | ? | Y |  |  |  |  |  |  |  | Y | ? | N | N | Y |  |  |  |  |  |  |  | Y | Y | Y | Y | N | 2 |
| Ishikawa et al., 2020 |  |  |  |  |  |  |  |  |  |  |  |  | Y | ? | Y | Y | ? |  |  |  |  |  |  |  |  |  |  |  |  | 3 |
| Kavanagh, 2019 | Y | Y | Y | Y | Y |  |  |  |  |  |  |  |  |  |  |  |  |  |  |  |  |  |  |  |  |  |  |  |  | 5 |
| King et al., 2021 | Y | ? | Y | Y | ? |  |  |  |  |  |  |  |  |  |  |  |  |  |  |  |  |  |  |  |  |  |  |  |  | 3 |
| King, 2016 | Y | N | Y | Y | N |  |  |  |  |  |  |  | ? | N | ? | N | Y |  |  |  |  |  |  |  | Y | Y | Y | N | N | 1 |
| Kirk, 2019 | Y | Y | Y | Y | Y |  |  |  |  |  |  |  |  |  |  |  |  |  |  |  |  |  |  |  |  |  |  |  |  | 5 |
| McCullagh, 2020 |  |  |  |  |  |  |  |  |  |  |  |  | Y | Y | Y | Y | Y |  |  |  |  |  |  |  |  |  |  |  |  | 5 |
| Moore, 2014 | Y | ? | Y | ? | Y |  |  |  |  |  |  |  |  |  |  |  |  |  |  |  |  |  |  |  |  |  |  |  |  | 3 |
| Moreno, 2019 |  |  |  |  |  |  | Y | Y | Y | Y | Y |  |  |  |  |  |  |  |  |  |  |  |  |  |  |  |  |  |  | 5 |
| Mudge, 2015 | ? | N | ? | ? | N |  |  |  |  |  |  |  |  |  |  |  |  |  | N | N | N | N | ? |  | N | N | N | N | N | 0 |
| O'Hare, 2017 | Y | Y | ? | Y | Y |  |  |  |  |  |  |  |  |  |  |  |  |  |  |  |  |  |  |  |  |  |  |  |  | 4 |
| Pavon et al., 2020 | Y | Y | Y | Y | Y |  |  |  |  |  |  |  |  |  |  |  |  |  |  |  |  |  |  |  |  |  |  |  |  | 5 |
| Pedersen et al., 2020 | Y | Y | Y | Y | Y |  |  |  |  |  |  |  |  |  |  |  |  |  |  |  |  |  |  |  |  |  |  |  |  | 5 |
| Porserud, 2019 |  |  |  |  |  |  |  |  |  |  |  |  | Y | Y | Y | N | Y |  |  |  |  |  |  |  |  |  |  |  |  | 4 |
| Resnick, 2015 |  |  |  |  |  |  |  |  |  |  |  |  |  |  |  |  |  |  | N | Y | N | ? | Y |  |  |  |  |  |  | 2 |
| S. Lim, 2020 | Y | Y | ? | ? | N |  |  |  |  |  |  |  | N | Y | Y | N | Y |  |  |  |  |  |  |  | Y | Y | Y | N | N | 2 |
| S.H. Lim, 2020 | Y | Y | Y | Y | Y |  |  |  |  |  |  |  |  |  |  |  |  |  |  |  |  |  |  |  |  |  |  |  |  | 5 |
| Said et al., 2021 |  |  |  |  |  |  |  |  |  |  |  |  | Y | N | Y | Y | Y |  |  |  |  |  |  |  |  |  |  |  |  | 4 |
| Scheerman et al., 2020 | Y | Y | Y | N | N |  |  |  |  |  |  |  |  |  |  |  |  |  | Y | Y | N | ? | N |  | N | N | N | N | N | 2 |
| Scheerman et al., 2021 | Y | Y | ? | ? | N |  |  |  |  |  |  |  |  |  |  |  |  |  | Y | Y | N | ? | N |  | N | N | N | N | N | 2 |
| Shannon, 2019 |  |  |  |  |  |  |  |  |  |  |  |  | N | N | ? | N | Y |  |  |  |  |  |  |  |  |  |  |  |  | 1 |
| So, 2012 | Y | Y | Y | Y | Y |  |  |  |  |  |  |  |  |  |  |  |  |  |  |  |  |  |  |  |  |  |  |  |  | 5 |
| Stefánsdóttir et al., 2021 | Y | Y | Y | Y | Y |  |  |  |  |  |  |  |  |  |  |  |  |  |  |  |  |  |  |  |  |  |  |  |  | 5 |
| Sun, 2020 | Y | N | Y | Y | N |  |  |  |  |  |  |  |  |  |  |  |  |  |  |  |  |  |  |  |  |  |  |  |  | 3 |
| Tousignant-Laflamme, 2015 | Y | ? | Y | N | N |  |  |  |  |  |  |  | N | Y | ? | N | N |  |  |  |  |  |  |  | Y | Y | Y | N | N | 1 |
| Van der Sluis, 2015 | N | Y | ? | ? | N |  |  |  |  |  |  |  | Y | Y | N | Y | Y |  |  |  |  |  |  |  | N | Y | Y | Y | N | 1 |
| Zisberg, 2016 |  |  |  |  |  |  |  |  |  |  |  |  | Y | N | N | Y | ? |  |  |  |  |  |  |  |  |  |  |  |  | 2 |
| Zisberg, 2018 | Y | ? | N | N | N |  |  |  |  |  |  |  | Y | Y | ? | N | Y |  |  |  |  |  |  |  | Y | Y | ? | N | N | 1 |

The overall quality score reflects the number of criteria satisfied, varying from no criteria met to all criteria met (5). For mixed method studies, there are 15 criteria to rate (instead of 5) and the overall quality score is the lowest score of these study components; Y = yes/good, N = no/not good, ? = can't tell/not sure; - = no quality criteria is met; underlined articles are mixed method studies; RCT = Randomized Controlled Trials; RQ = research question

1.1 = Is the qualitative approach appropriate to answer the RQ?; 1.2 = 1.2. Are the qualitative data collection methods adequate to address the RQ?; 1.3 = Are the findings adequately derived from the data?; 1.4 = Is the interpretation of results sufficiently substantiated by data?; 1.5 = Is there coherence between qualitative data sources, collection, analysis and interpretation?; 2.1 = Is randomization appropriately performed?; 2.2 = Are the groups comparable at baseline?; 2.3 = Are there complete outcome data?; 2.4 = Are outcome assessors blinded to the intervention provided?; 2.5 = Did the participants adhere to the assigned intervention?; 3.1 = Are the participants representative of the target population?; 3.2 = Are measurements appropriate regarding both the outcome and intervention/exposure?; 3.3 = Are there complete outcome data?; 3.4 = Are the confounders accounted for in the design and analysis?; 3.5 = During the study period, is the intervention/exposure administered as intended? 4.1 = Is the sampling strategy relevant to address the RQ?; 4.2 = Is the sample representative of the target population?; 4.3 = Are the measurements appropriate?; 4.4 = Is the risk of nonresponse bias low?; 4.5 = Is the statistical analysis appropriate to answer the research question?; 5.1 = Is there an adequate rationale for using a mixed methods design to address the RQ?; 5.2 = Are the different components of the study effectively integrated to answer the RQ?; 5.3 = Are the outputs of the integration of qualitative and quantitative components adequately interpreted?; 5.4 = Are divergences and inconsistencies between quantitative and qualitative results adequately addressed?; 5.5 = Do the different components of the study adhere to the quality criteria of each tradition of the methods involved?;
