# Supplementary material for: Facilitators and barriers to enhancing physical activity in older patients during acute hospital stay: a systematic review
Source: Int J Behav Nutr Phys Act. 2022 Jul 30;19:99. doi: 10.1186/s12966-022-01330-z (PMC9338465; doi:10.1186/s12966-022-01330-z)
Supplement: Supplementary file 7 — Additional file 7. Overview of facilitators and barriers per (sub)theme. Table presenting an overview of all identified facilitators and barriers per (sub)theme and per perspective. [file 12966_2022_1330_MOESM7_ESM.docx]

**Additional file 7.** Overview of facilitators and barriers in relation to (sub)themes within the social-ecological model. Described by the three perspectives of patient, caregiver and health care professional.

| **Facilitator^a^** | |  | **Barrier^a^** | |
| --- | --- | --- | --- | --- |
| **Quantitative studies** | **Qualitative studies** |  | **Quantitative studies** | **Qualitative studies** |
| **Patient perspective** | | | | |
| **Intrapersonal level** | | | | |
| **1. Patients' knowledge, awareness and attitude** | | | | |
| **1.1 Knowledge, awareness, attitude** | | | | |
| *8* | *24* |  | *6* | *19* |
| Positive attitude (Zisberg et., al 2018) | Experience with negative consequences of inactivity and long bed rest episodes (De Klein et al., 2019; Stefánsdóttir et al., 2021) |  | Negative/neutral attitude* (OR = 3.22, 95% CI: 1.24-8.44) (Zisberg et al., 2018) | Expectations: in general (S. Lim et al., 2020); negative pre-existing (O'hare et al., 2017); and hospital admission is not associated with PA (De Klein et al., 2019) |
| More satisfied with hospital physical environment* (OR = 1.48, 95% CI: 1.00-2.17, p < .05) (Zisberg et al., 2016) | Positive pre-existing expectations (O'hare et al., 2017) |  | Agreeing with stay in bed when I'm sick (Zisberg et al., 2018) | Lack of control (Boltz et al., 2010) |
| Physical activity would contribute to recovery (Scheerman et al., 2021) | Positive experience (S. Lim et al., 2020) |  | Unwillingness to move (Moreno et al., 2019) | Preference of staying in bed: is more comfortable (De Klein et al., 2019); and bedrest to aid recovery (S.H. Lim et al., 2020) |
| Physical activity during admission is important (Scheerman et al., 2021) | Promotion of functional recovery (So et al., 2012) |  | Patient refusal (Hamilton et al., 2019) | Little information about PA during admission (De Klein et al., 2019) |
| Maintenance of strength (Scheerman et al., 2021) | Avoiding further loss of function (Mudge et al., 2015) |  | Disagree with walking will help to maintain pre-hospital function (Zisberg et al., 2018) | Lack of motivation (Sun et al., 2020; Stefánsdóttir et al., 2021) |
| PA has a positive influence on self-confidence (Scheerman et al., 2021) | Awareness of the need for mobility (S.H. Lim et al., 2020; Andreasen et al., 2018; King et al., 2021) |  | Rest would contribute to recovery (Scheerman et al., 2021) | Not accustomed to PA (Sun et al., 2020) |
| Mood (Scheerman et al., 2021) | Patient knowledge (O'hare et al., 2017) |  |  | Unfamiliar environment or surroundings (S.H. Lim et al., 2020; So et al., 2012; King et al., 2021) |
| Feeling physically independent when being physically active (Scheerman et al., 2021) | Not wanting to rely on others (Mudge et al., 2015) |  |  | Sense of loss of mobility (Haslam-Larmer et al., 2021) |
|  | Recognized the contribution of tasks to expediting discharge home (Mudge et al., 2015) |  |  | Feelings of losing autonomy (Pavon et al., 2021) |
|  | Doing activities for symptom management (Mudge et al., 2015) |  |  | Feeling isolated, e.g. physical, restricted to their room; and psychological, not feeling engaged or part of the healthcare team (King et al., 2021) |
|  | Preventing boredom (Mudge et al., 2015) |  |  | Uncertain about ambulation (King et al., 2021) |
|  | Perceived benefits (S. Lim et al., 2020; Sun et al., 2020) or positive outcomes (O'hare et al., 2017) |  |  | Physical appearance: looking disheveled, weak, or struggling (King et al., 2021) |
|  | Improved well-being (So et al., 2012) |  |  | Boredom (Scheerman et al., 2021) |
|  | Avoiding negative effects of bed rest, e.g. boredom, pain, functional decline, fatigue, inactivity (So et al., 2012) |  |  |  |
|  | Need to care for themselves by getting up and maintaining or building strength (King et al., 2021) |  |  |  |
|  | The need to resume 'normal' life for quick recovery (Haslam-Larmer et al., 2021) |  |  |  |
|  | Physical appearance: having enough time to groom and dress (King et al., 2021) |  |  |  |
|  | Motivated to return to meaningful activities outside the hospital (Stefánsdóttir et al., 2021) |  |  |  |
|  | Motivated to reach goals or attend activities during hospitalization (Stefánsdóttir et al., 2021) |  |  |  |
| **2. Patients' personal and health factors** | | | | |
| **2.1 Patients' personal factors** | | | | |
| *2* | *0* |  | *5* | *0* |
| Younger age* (OR = .92, 95% CI: 0.88-0.96, p < .001) (Zisberg et al., 2016) |  |  | Younger age: age 65-74* (OR = 1.68, 95% CI: 1.15-2.47, p = .008) and age 75-84* (OR = 2.19, 95% CI: 1.50-3.20, p < 0.001) (Chase et al., 2018) |  |
| Males* (OR = 1.88, 95% CI: 1.25-2.76, p < .001) (Zisberg et al., 2016) |  |  | Age ≥ 75 years* (OR = 4,47, 95% CI: 1.38-14.45, p = .012) (Agostini et al., 2014) |  |
|  |  |  | Older age* (r = -.42, p = .04) (Resnick et al., 2015) |  |
|  |  |  | Non-white ethnicity* (p = .026) (Fisher et al., 2011) |  |
|  |  |  | Age > 65 years* (OR = 2.49, 95% CI: 1.04-6.09, p = .039) (Ishikawa et al., 2020) |  |
| **2.2 Emotional status** | | | | |
| *1* | *1* |  | *5* | *9* |
| Less anxiety symptoms* (OR = .62, 95% CI: 0.39-0.97, p < .05) (Zisberg et al., 2016) | Coping strategies to counter anxiety and stress (Sun et al., 2020) |  | Fear of infection (Moreno et al., 2019) | Fear of falling (Boltz et al., 2010; Brown et al., 2007; S.H. Lim et al., 2020; Pavon et al., 2021) |
|  |  |  | Fear of falling (Moreno et al., 2019; Scheerman et al., 2021) | Fear of burden to nurse when falling while ambulating alone (S.H. Lim et al., 2020) |
|  |  |  | Fear of missing a doctor's visit (Moreno et al., 2019) | Fear of injury (So et al., 2012) |
|  |  |  | Fear of losing venous access (Moreno et al., 2019) | Anxiety / feeling overwhelmed (Sun et al., 2020) |
|  |  |  |  | Fear of falling, fear of pain, or fear of reinjury (Haslam-Larmer et al., 2021) |
|  |  |  |  | Concerns of causing problems, e.g. dislodging stitches, feeling physically drained, increased pain or becoming too weak (King et al., 2021) |
| **2.3 Cognitive status** | | | | |
| *1* | *0* |  | *2* | *0* |
| Higher cognitive status* (OR = 1.14, 95% CI: 1.02-1.26, p < .05) (Zisberg et al., 2016) |  |  | Neuropsychiatric symptoms or apathy* (p < .001) (Belala et al., 2019) |  |
|  |  |  | More symptoms of depression* (r = -.45, p = .03) (Resnick et al., 2015) |  |
| **2.4 Physical health** | | | | |
| *8* | *0* |  | *12* | *14* |
| Less risk of malnutrition* (OR = .74, 95% CI: 0.72-0.95, p < .05) (Zisberg et al., 2016) |  |  | Having a hearing impairment* (OR = 1.58, 95% CI: 1.12-2.23, p < .009) (Chase et al., 2018) | Having various symptoms (Brown et al., 2007; So et al., 2012; Boltz et al., 2010; Sun et al., 2020), e.g. weakness, pain, fatigue, dyspnea, shortness of breath, nausea, dizziness |
| Higher premorbid physical activity level (YPAS)* (OR = 1.01, 95% CI: 1.00-1.02, p < .05) (Zisberg et al., 2016) |  |  | Having various symptoms (Moreno et al., 2019; S. Lim et al., 2020; Haines et al., 2013; Agostini et al., 2014; Scheerman et al., 2021), e.g. dyspnea, pain, dizziness, tiredness, feeling ill, cardiac instability, drowsiness, hypotension | Patients poor physical health status (De Klein et al., 2019; O'hare et al., 2017) |
| Higher baseline physical performance (SPPB)* (1.15, 95% CI: 1.08-1.22) (McCullagh et al., 2020); increase in SPPB* (p = .048) (Evensen et al., 2017) |  |  | Predicted FEV1 < 70%* (OR = 2.72, 95% CI: 1.08-6.81, p = .033) (Agostini et al., 2014) | Patient's need for assistance to ambulate (Brown et al., 2007) |
| Higher baseline Barthel index* (OR = 1.03, 95% CI: 1.01-1.04, p < .001) (Zisberg et al., 2016) |  |  | Lower preoperative activity level* (OR = 3.73, 95% CI: 1.24-11.25, p = .019) (Agostini et al., 2014) | Comorbid conditions, procedures and injuries (Sun et al., 2020) |
| Higher pre-fracture functional status score (NMS)* (p = .04) (Haslam-Larmer et al., 2021) |  |  | Dependent or with assistance prior mobility status* (p < .001) (Fisher et al., 2011) | Functional limitations/history of falls (Sun et al., 2020) |
| Higher pre-fracture functional status score (NMS)* (OR = 1.30, 95% CI: 1.06-1.60, p = .012) (Said et al., 2021) |  |  | Low pre-fracture functional status and cognitive impairment (MMSE < 24) (Haslam-Larmer et al., 2021) | Impaired physical condition (Andreasen et al., 2018) |
| Higher level of functional mobility ability (DEMMI) (Hartley et al., 2020) |  |  | Increase in blood urea nitrogen (BUN)* (OR = 1.04, 95% CI: 1.01-1.08, p = .002) (Ishikawa et al., 2020) | Patient health status (Pavon et al., 2021) |
|  |  |  | Postoperative delirium* (OR = .25, 95% CI: 0.08-0.79, p = .019) (Said et al., 2021) | Varying acute medical conditions (on day 1 or day 2), e.g. pain, blood transfusion, drowsiness, low blood pressure (Haslam-Larmer et al., 2021) |
|  |  |  |  | Illness experience of symptoms, such as pain and weakness (Stefánsdóttir et al., 2021) |
|  |  |  |  | Fatigue (Stefánsdóttir et al., 2021) |
| **3. Medical-related factors** | | | | |
| **3.1 Presence of lines/attachments** | | | | |
| *1* | *0* |  | *4* | *5* |
| More tethers* (r = .49, p = .03) (Resnick et al., 2015) |  |  | Devices or treatments that hinder walking* (.71, 95% CI: 0.56-0.91) (McCullagh et al., 2020) | Presence of medical devices (De Klein et al., 2019) |
|  |  |  | Use of intravenous medications (Moreno et al., 2019) | Tethers (So et al., 2012) |
|  |  |  | Having lines or drains (Scheerman et al., 2021) | Having intravenous line or urinary catheter (Brown et al., 2007) |
|  |  |  | Indwelling catheter* (RR = 1.38, 95% CI: 1.09-1.73, p = .006) (Chua et al., 2017) | Intravenous lines attached to pumps and urinary catheters (King et al., 2021) |
|  |  |  |  | Lines or drains (Scheerman et al., 2021) |
| **3.2 Admitting diagnosis and illness severity** | | | | |
| *4* | *0* |  | *3* | *1* |
| Lower illness severity* (OR = .93, 95% CI: 0.88-0.99, p < 05) (Zisberg et al., 2016) |  |  | Major illness severity* (p = .011) (Fisher et al., 2011) | Illness or surgery (Scheerman et al., 2021) |
| Improving medical status* (1.33, 95% CI: 1.07-1.64) (McCullagh et al., 2020) |  |  | Diagnostic category: orthopedic / musculoskeletal* (p = .001) (Fisher et al., 2011) |  |
| No acute complications* (RR = 1.20, 95% CI: 1.04-1.38, p = .013) (Chua et al., 2017) |  |  | Non-emergency admit* (OR = 1.62, 95% CI: 1.06-2.49, p < .026) (Chase et al., 2018) |  |
| Lower illness severity (lower admission CRP and NEWS) (Hartley et al., 2020) |  |  |  |  |
| **3.3 Treatment-related factors** | | | | |
| *3* | *0* |  | *3* | *2* |
| Less in-hospital sleep medication* (OR = 1.99, 95% CI: 1.03-3.85, p < .05) (Zisberg et al., 2016) |  |  | Presence of bedrest order* (0.31, 95% CI: 0.21-0.45) (McCullagh et al., 2020) | Adverse events, e.g. wrong medication or infections (Boltz et al., 2010) |
| Higher daily caloric intake* (OR = 2.61, 95% CI: 1.27-5.95) (Zisberg et al., 2016) |  |  | Longer length of stay* (OR = 1.04, 95% CI:1.00-1.08, p = .035) (Chase et al., 2018) | Prescribed immobility (Boltz et al., 2010) |
| Absence of donor blood transfusion* (RR = 1.29, 95% CI: 1.03-1.63, p = .028) (Chua et al., 2017) |  |  | Continues oxygen therapy (Moreno et al., 2019) |  |
| **Interpersonal level** | | | | |
| **4. Social support** | | | | |
| **4.1 Patient - informal network** | | | | |
| *0* | *5* |  | *3* | *1* |
|  | Identification and support of family or friends (Boltz et al., 2010) |  | Patient alone* (p < .001) (Belala et al., 2019) | Visitors (Andreasen et al., 2018) |
|  | Social aspect of intervention (S. Lim et al., 2020) |  | Presence of relatives / friends* (p < .001) (Belala et al., 2019) |  |
|  | Social / family support (Sun et al., 2020) |  | Lack of companion encouragement (Moreno et al., 2019) |  |
|  | Social stimulation with other patients (King et al., 2021) |  |  |  |
|  | Motivated by relatives to increase mobility (Stefánsdóttir et al., 2021) |  |  |  |
| **4.2 Patient - HCP** | | | | |
| *3* | *13* |  | *1* | *18* |
| Presence of service staff* (p < .001) (Belala et al., 2019) | Nurses have attention to comfort and hygiene (Boltz et al., 2010) |  | Lack of professional help (Moreno et al., 2019) | Lack of encouragement or support from HCP (So et al., 2012; King et al., 2021) |
| Presence of therapist* (p < .001) and nurses* (p < .05) (Belala et al., 2019) | Positive bedside manner of nurses (Boltz et al., 2010) |  |  | Discouraged by HCP (So et al., 2012) |
| Being asked to be physically active (Scheerman et al., 2021) | HCP asked patients to exercise (So et al., 2012) |  |  | Staff not knowing the person (Boltz et al., 2010) |
|  | Encouragement from HCP (Boltz et al., 2010) |  |  | Absence of physiotherapist as a guide to exercise (Andreasen et al., 2018) |
|  | Presence of dietician and physiotherapists (Andreasen et al., 2018) |  |  | Patient felt not being seen by HCP (Andreasen et al., 2018) |
|  | Patient felt seen and believed in by HCP (Andreasen et al., 2018) |  |  | Patients don't want to bother nurses / staff (Brown et al., 2007; Stefánsdóttir et al., 2021) |
|  | Perceived professionalism of HCP (O'hare et al., 2017) |  |  | Staff is lacking interest of mobility importance (Brown et al., 2007) |
|  | Perceived authority figure of HCP (O'hare et al., 2017) |  |  | Inconsistent and confusing advice and assistance of HCPs on mobility (Pavon et al., 2021) |
|  | Perceived personality of HCP (O'hare et al., 2017) |  |  | Patient not being told how to handle machines or tubes (King et al., 2021) |
|  | Encouragement by HCPs, especially by physicians (Scheerman et al., 2021) |  |  | Permission of HCP to walk (King et al., 2021) |
|  | HCPs providing information about walking (King et al., 2021) |  |  | Timing and duration of mobility activities is up to HCP (Haslam-Larmer et al., 2021) |
|  | HCPs encouragement and praise (Stefánsdóttir et al., 2021) |  |  | Patients don't asked to go for a walk by the appearance of nurses being busy (King et al., 2021) |
|  | HCPs having time to assist walking (Stefánsdóttir et al., 2021) |  |  | HCPs limited communication about walking (King et al., 2021) |
|  |  |  |  | Patients waited until nurses initiated the conversation about walking (King et al., 2021) |
|  |  |  |  | HCPs concerns about falls (King et al., 2021) |
|  |  |  |  | Safety concerns of HCPs on patient moving (Pavon et al., 2021) |
| **Institutional level** | | | | |
| **5. Physical environment** | | | | |
| **5.1 Space and location** | | | | |
| *2* | *6* |  | *3* | *7* |
| Hallway or bathroom* resulting in higher activity difficulty level (p < .001) (Belala et al., 2019) | The right equipment, e.g. handrails in patient room (Boltz et al., 2010) |  | Patient room* resulting in lower activity level (p < .001) (Belala et al., 2019) | High beds (Boltz et al., 2010) |
| Patient room with family space* (Wilcoxon Rank Sum test z = -3.28, p = .001) (Shannon et al., 2019) | Adjustments in the hospital environment (De Klein et al., 2019) |  | Outside the patient room in communal spaces (Shannon et al., 2019) | Non-welcoming environments for walking (King et al., 2021) |
|  | Physical ward environment, e.g. exercise bicycles, marked ambulation route in hallway (Andreasen et al., 2018) |  | Lack of space (Moreno et al., 2019) | No wayfinding maps in the unit or building to orient (King et al., 2021) |
|  | Improving and enlarging the areas for group activities (Feenstra et al., 2021) |  |  | Clutter in room or hallways, and patients rooms small and cramped (King et al., 2021) |
|  | Niches in corridor giving patients option to take a break (Feenstra et al., 2021) |  |  | Danger zones' in patient room or hallways, where patients are unable to get help if needed or are unable to get back to their room due to fatigue (King et al., 2021) |
|  | Widening the living room and adding flexibility (Feenstra et al., 2021) |  |  | Limited seating or no optimal seating (King et al., 2021) |
|  |  |  |  | Uninviting design and furnishing of the department (Stefánsdóttir et al., 2021) |
| **6. Resources** | | | | |
| **6.1 Staffing** | | | | |
| *0* | *0* |  | *0* | *4* |
|  |  |  |  | Insufficient staff (Boltz et al., 2010) |
|  |  |  |  | Shortage of staff (Brown et al., 2007; S.H. Lim et al., 2020) |
|  |  |  |  | Busy staff (Stefánsdóttir et al., 2021) |
| **6.2 Time and competing priorities** | | | | |
| *0* | *0* |  | *0* | *2* |
|  |  |  |  | Lack of time of staff (S.H. Lim et al., 2020; Brown et al., 2007) |
| **6.3 Equipment** | | | | |
| *0* | *0* |  | *2* | *2* |
|  |  |  | Lack of equipment (Moreno et al., 2019) | Lack of assistive devices (So et al., 2012) |
|  |  |  | Absence of assistive devices (Scheerman et al., 2021) | No means to alert staff if patients need help (King et al., 2021) |
| **6.4 Education and information** | | | | |
| *0* | *1* |  | *0* | *1* |
|  | Staff education (Boltz et al., 2010) |  |  | Invisible posters and folders encouraging in-hospital mobility (Stefánsdóttir et al., 2021) |
| **7. Organizational factors** | | | | |
| **7.1 Hospital routines and activities** | | | | |
| *3* | *8* |  | *2* | *4* |
| Activities of daily living and hospital routines* (p < .001) (Belala et al., 2019) | Preparation for discharge, e.g. walk to the bathroom instead use of urinal (Boltz et al. 2010) |  | Patient not available (Hamilton et al., 2019) | Wearing hospital gowns (Brown et al., 2007) |
| Hospital site* (p < .05) (Chua et al., 2017) | Keeping the mind active (Boltz et al., 2010) |  | Patients receiving nursing care or away for imaging or other treatment (S. Lim et al., 2020) | Patient not available (S. Lim et al., 2020) |
| Leisure time activities* (p < .001) (Belala et al., 2019) | Daily schedule or activity program (Boltz et al., 2010; De Klein et al., 2019) |  |  | Hospital gowns and restrictions to their undergarments (King et al., 2021) |
|  | Setting patient goals (Sun et al., 2020; O'hare et al., 2017) |  |  | Invisible PA intervention (walk plan) (Stefánsdóttir et al., 2021) |
|  | Meaningful PA intervention (walk plan) (Stefánsdóttir et al., 2021) |  |  |  |
|  | Option of self-service on beverages and clothes (Stefánsdóttir et al., 2021) |  |  |  |
|  | Shared meals with other patients (Feenstra et al., 2021) |  |  |  |
| **7.2 Daytime or weekday** | | | | |
| *2* | *0* |  | *5* | *0* |
| Morning* (p < .01) (Belala et al., 2019) |  |  | Whole afternoon* (p<.05) (Belala et al., 2019) |  |
| Wednesday* (1.26, 95% CI: 1.04-1.53) (McCullagh et al., 2020) |  |  | After lunch (1 p.m.)* (p < .001) (Belala et al., 2019) |  |
|  |  |  | First day of admission* (0.51, 95% CI: 0.42-0.62) (McCullagh et al., 2020) |  |
|  |  |  | Postoperative day 1, having symptoms (divers) (Haines et al., 2013; Agostini et al., 2014) |  |
|  |  |  | Postoperative day 2, having symptoms (pain) (Agostini et al., 2014) |  |
| **7.3 Rules, regulations and policies** | | | | |
| *0* | *1* |  | *0* | *1* |
|  | Set standards to be active (e.g. administration) (Boltz et al., 2010) |  |  | Messaging about restricted movement, e.g. orally, visually by signs or auditory by motion sensors (King et al., 2021) |
| **Caregiver perspective** | | | | |
| **Intrapersonal level** | | | | |
| **8. Caregiver knowledge, awareness, and attitude** | | | | |
| **8.1 Knowledge, awareness, and attitude** | | | | |
| *n/a* | *3* |  | *n/a* | *4* |
|  | Providing support to patient (Sun et al., 2020) |  |  | Perceived importance of bed rest to aid recovery (S.H. Lim et al., 2020) |
|  | Awareness of the need for mobility (S.H. Lim et al., 2020) |  |  | Anxiety/feeling overwhelmed (Sun et al., 2020) |
|  | Seeing benefits from walking (Sun et al., 2020) |  |  | Patient is not accustomed to physical activity (Sun et al., 2020) |
|  |  |  |  | Lack of motivation (Sun et al., 2020) |
| **8.2 Patient safety concerns** | | | | |
| *n/a* | *0* |  | *n/a* | *2* |
|  |  |  |  | Fear of patient falling (S.H. Lim et al., 2020) |
|  |  |  |  | Fear of burden to nurse when patient falls (S.H. Lim et al., 2020) |
| **Interpersonal level** | | | | |
| **9. Patients health status and medical-related factors** | | | | |
| **9.1 Physical or mental health** | | | | |
| *n/a* | *0* |  | *n/a* | *2* |
|  |  |  |  | Patients comorbid conditions, procedures and injuries (Sun et al., 2020) |
|  |  |  |  | Patient is having physical symptoms, e.g. pain, dyspnea, fatigue (Sun et al., 2020) |
| **Institutional level** | | | | |
| *n/a* | *0* |  | *n/a* | *0* |
| **Healthcare professional perspective** | | | | |
| **Intrapersonal level** | | | | |
| **10. HCP knowledge, awareness and attitude** | | | | |
| **10.1 Knowledge, awareness and attitude** | | | | |
| *4* | *11* |  | *5* | *15* |
| Physical therapist have highest knowledge and most positive attitudes (Zisberg et al., 2018) | Knowledge: not specified (Andreasen et al., 2018); of patients condition to assess ambulation status (S.H. Lim et al., 2020) |  | Nurses knowledge towards patients walking in hall*(p < .01) (Dermody et al., 2018) | Lack of knowledge: informing and motivating (De Klein et al., 2019); mobilization techniques (Moore et al., 2014; Boltz et al., 2011); psychosocial needs (Boltz et al., 2011); the intervention (Andreasen et al., 2018); patients mobility status (Moore et al., 2014); immobilization syndrome and consequences (Tousignant-Laflamme et al., 2015); safety assessment (Zisberg et al., 2018) |
| Knowledge of methods of PA promotion (Scheerman et al., 2020) | Change of mindset that PA is important for recovery, next to medicine (De Klein et al., 2019) |  | Nurses attitude towards patients walking in hall*(p < .035) (Dermody et al., 2018) | Ambulation is ideal, but not necessary (Doherty-King et al., 2011) |
| Knowledge of importance of PA promotion (Scheerman et al., 2020) | Awareness of the need for mobility (S.H. Lim et al., 2020) |  | RN, NA and MD have poor knowledge and negative attitude (Zisberg et al., 2018) | PA is not yet seen as part of usual hospital care by HCPs (De Klein et al., 2019) |
| Motivation of nurses (Scheerman et al., 2020) | Perceived benefits by staff (S. Lim et al., 2020) |  | Ward staff is not adequate to mobilize (Zisberg et al., 2018) | Lack of awareness of the intervention (S. Lim et al., 2020) |
|  | Patient benefits (S. Lim et al., 2020; Mudge et al., 2015) |  | RN, NA and MD have low knowledge on safe mobilization training (Zisberg et al., 2018) | Staff resistance to program (Kavanagh et al., 2019) |
|  | Positive experience by volunteers (S. Lim et al., 2020) |  | Feeling uncertain when it was safe to promote mobility (Dermody et al., 2017) | Resistance towards health promotion (Andreasen et al., 2018) |
|  | Motivated to prevent risk of complications, loss of muscle mass and slower recovery (Scheerman et al., 2020) |  |  | Doubting about own ability to perform rehabilitation tasks (Van der Sluis et al., 2015) |
|  | Awareness of hospital-based policies to promote early mobilization (Haslam-Larmer et al., 2021) |  |  | Need for PA promotion in older patients is questioned (Scheerman et al., 2020) |
|  | Awareness of the negative consequences of inactivity during hospitalization (Pedersen et al., 2020) |  |  |  |
| **10.2 Patient safety concerns** | | | | |
| *0* | *0* |  | *5* | *3* |
|  |  |  | Mobility may pose a greater risk for injury (Dermody et al., 2017) | Fear of patient falling (Boltz et al., 2011; Brown et al., 2007) |
|  |  |  | Mobility could be harmful (Dermody et al., 2017) | Fears of injuries to patient or themselves (Moore et al., 2014) |
|  |  |  | Safety concerns (Babine et al., 2019) |  |
|  |  |  | Liability concerns (Babine et al., 2019) |  |
|  |  |  | Risk of staff or patient injury while performing tasks (Said et al., 2021) |  |
| **11. HCP expertise and characteristics** | | | | |
| **11.1 Expertise and characteristics** | | | | |
| *1* | *4* |  | *5* | *1* |
| Nurses with less experience were more confident in mobilizing patients* (p = .009) (Dermody et al., 2017) | Experience from geriatric specialization (Kirk et al., 2019) |  | Nurses with less experience were less likely to view mobility promotion as priority* (p = .024) (Dermody et al., 2017) | Nurses lacking physical strength to mobilize high-risk patient (Doherty-King et al., 2011) |
|  | Experience in long-term care settings or rehabilitation (Doherty-King et al., 2011) |  | Nurses with less experience have less knowledge on items: receipt of training* (p = .004), when to refer to physical therapy* (p = .047), when to refer to occupational therapy* (p = .007) (Dermody et al., 2017) |  |
|  | HCP competence (S. Lim et al., 2020) |  | Lack of confidence (Dermody et al., 2017) |  |
|  | Strong and confident nurses (Doherty-King et al., 2011) |  |  |  |
| **Interpersonal level** | | | | |
| **12. Patient cooperation** | | | | |
| **12.1 Patient - informal network** | | | | |
| *0* | *5* |  | *0* | *3* |
|  | Strong family support system (Chan et al., 2019) |  |  | Lack of practical assistance from relatives (Kirk et al., 2019) |
|  | Family (Doherty-King et al., 2011) |  |  | Family beliefs that patient need to rest (Boltz et al., 2011) |
|  | Verbalization and practical assistance from relatives (Kirk et al., 2019) |  |  | Patient is encouraged by family to rest and remain in bed (Boltz et al., 2011) |
|  | Include the family in care provision (Boltz et al., 2011) |  |  |  |
|  | Empower and enhance role family members in patients care (S.H. Lim et al., 2020) |  |  |  |
| **12.2 Patient - HCP** | | | | |
| *1* | *15* |  | *3* | *27* |
| PA promotion by physician (Scheerman et al., 2020) | Community patients (Doherty-King et al., 2011) |  | Patients declined to mobilize (Dermody et al., 2017; Said et al., 2021) | Patients not aware of effects of long bed rest episodes (De Klein et al., 2019) |
|  | Knowing patients functional ability (Chan et al., 2019) |  | Patient preference of HCP's mobility promotion (Dermody et al., 2018) | Patients adoption of sick-role behavior (Chan et al., 2019; Moore et al., 2014) |
|  | Patients anticipated to mobilize in hospital (Kirk et al., 2019) |  |  | Patients need for assistance (Brown et al., 2007) |
|  | Communication: aging-sensitive (Boltz et al., 2011) |  |  | Patients baseline is non ambulatory, even incorrectly (Doherty-King et al., 2011) |
|  | Nurses understand patients to motivate them (Boltz et al., 2011) |  |  | Nursing home patients (Doherty-King et al., 2011) |
|  | Nurses showing caring and kindness (Boltz et al., 2011) |  |  | Patients find it a privilege to stay in bed (Kirk et al., 2019) |
|  | Drawing social contracts between HCP and patient (Chan et al., 2019) |  |  | Patients wearing pajamas during the day (De Klein et al., 2019) |
|  | Mobility encouragement by physiotherapists (Kirk et al., 2019), or by physician (Pedersen et al., 2020) |  |  | Patients fear of falling (Boltz et al., 2011; Moore et al., 2014) |
|  | Physiotherapist in 'sporty clothes' (Kirk et al., 2019) |  |  | Patients being quiet (Boltz et al., 2011) |
|  | Support of nurse specialists (Doherty-King et al., 2011) |  |  | Communication: language (Boltz et al., 2011; Chan et al., 2019) |
|  | Promotion of patient self-care (Boltz et al., 2011) |  |  | Lack of patient motivation (Brown et al., 2007; Moore et al., 2014) |
|  | HCP emphasizing the importance of ambulation with patients and family (King et al., 2016) |  |  | Patient is over-relianced on domestic helper (Chan et al., 2019) |
|  | Motivating patients to be active (Scheerman et al., 2020) |  |  | Patient's social suppositions on paid services (Chan et al., 2019) |
|  | Rewards for patients to leave their bed, e.g. such as TV in seating area (Feenstra et al., 2021) |  |  | HCP telling unintentionally how comfortable the bed is (De Klein et al., 2019) |
|  |  |  |  | HCP servicing self-supporting patients (Kirk et al., 2019) |
|  |  |  |  | Patients expected that nurses have not the same responsibility for mobilization (Kirk et al., 2019) |
|  |  |  |  | Encouragement by nurses or nursing assistants (Kirk et al., 2019) |
|  |  |  |  | Patients do not cooperate with instructions to mobilize (Moore et al., 2014) |
|  |  |  |  | Patients have little knowledge of importance of early mobility (Moore et al., 2014) |
|  |  |  |  | Patients fear of falling, fear of pain, or fear of reinjury (Haslam-Larmer et al., 2021) |
|  |  |  |  | Patients perception of hospital as a place to rest, be sick, and being physically inactive (Scheerman et al., 2020) |
|  |  |  |  | Patient motivation (Scheerman et al., 2020) |
|  |  |  |  | HCPS empathic towards patients facilitating comfort rather than PA (Scheerman et al., 2020) |
| **13. Clinician and team influences** | | | | |
| **13.1 Collaboration** | | | | |
| *1* | *14* |  | *1* | *4* |
| Nurses consult other HCPs to promote PA (Scheerman et al., 2020) | Collaboration: not specified (Kavanagh et al., 2019); among team (Boltz et al., 2011); between staff, project team and patients (Andreasen et al., 2018) |  | HCPs discussion of patients mobility level (Zisberg et al., 2018) | Lack of consistency and communication between HCP (Moore et al., 2014) |
|  | Engaging a multidisciplinary team approach (Chan et al., 2019) |  |  | Lack of communication between shifts (Tousignant-Laflamme et al., 2015) |
|  | Interprofessional collaboration between nursing staff and physiotherapists (Tousignant-Laflamme et al., 2015) |  |  | Lack of appreciation of staff (S. Lim et al., 2020) |
|  | Strong teamwork and willingness to adopt new practices (Zisberg et al., 2018) |  |  | Communication among HCP by electronic health recording (Pavon et al., 2021) |
|  | Communication and case discussion (Moore et al., 2014) |  |  |  |
|  | Discussion of patient ambulation needs with HCP (King et al., 2016) |  |  |  |
|  | HCP feeling supported (King et al., 2016) |  |  |  |
|  | HCP getting support from staff change drivers (Kavanagh et al., 2019) |  |  |  |
|  | Presence of dietician and physiotherapists (Andreasen et al., 2018) |  |  |  |
|  | Appreciation of mobility volunteers (S. Lim et al., 2020) |  |  |  |
|  | Nurses seeing the physiotherapist on the ward (Tousignant-Laflamme et al., 2015) |  |  |  |
|  | Enhanced cross-professional cooperation focusing on mobility (Pedersen et al., 2020) |  |  |  |
| **13.2 Role clarity** | | | | |
| *2* | *3* |  | *0* | *8* |
| Nurses promote daily activities (Scheerman et al., 2020) | Extension of job description of nurses with tasks aimed at patient rehabilitation (Van der Sluis et al., 2015) |  |  | Lack of nurse autonomy (Boltz et al., 2011) or professional autonomy to intervene (Kavanagh et al., 2019) |
| Nurses promote additional PA like stretch and gait exercises (Scheerman et al., 2020) | Volunteers seen as non-medical personnel (S. Lim et al., 2020) |  |  | Unclear roles of nurses (Boltz et al., 2011) |
|  | Seeing physical activity as a fundamental facet of nursing (Chan et al., 2019) |  |  | Differences in medical specialties of HCP, e.g. work rhythms and procedures (Kirk et al., 2019) |
|  |  |  |  | HCP differences in which tasks, actions and language are considered important for influence patient mobility (Kirk et al., 2019) |
|  |  |  |  | Nurses, nurse assistants and physicians did not perceive mobilization as part of patient treatment or their core task (Kirk et al., 2019) |
|  |  |  |  | Different HCP sectors defined mobility differently, e.g. mobility dose, timing and monitoring (Zisberg et al., 2018) |
|  |  |  |  | Lack of clarity regarding roles (Pavon et al., 2021) |
| **13.3 Responsibility** | | | | |
| *2* | *7* |  | *0* | *8* |
| Nurses feel responsible to promote PA (Scheerman et al., 2020) | Interdisciplinary responsibility (Van der Sluis et al., 2015) |  |  | Unclear responsibilities (Boltz et al., 2011; Pavon et al., 2021) |
| Culture of PA promotion on ward (Scheerman et al., 2020) | Shifting responsibility towards nurse practioners (Van der Sluis et al., 2015) |  |  | Disagreement in responsibility (Zisberg et al., 2018) |
|  | Shifting ownership from physical therapy to nursing (King et al., 2016) |  |  | Lack of responsibility (Zisberg et al., 2018; Moore et al., 2014) |
|  | Local opinion leaders, e.g. mobility champions (Moore et al., 2014) |  |  | Unwillingness to accept extra workload (Pedersen et al., 2020) |
|  | HPCs feel responsible (De Klein et al., 2019) |  |  | Physicians do not focus on mobility and it's not their task (Pedersen et al., 2020) |
|  | Being responsible for signaling and performing PA promotion (Scheerman et al., 2020) |  |  | Mobility may not be a priority on all HCP services (Pavon et al., 2021) |
|  | Staff compromising with alternative mobility plan if unable to implement full mobility plan (Haslam-Larmer et al., 2021) |  |  |  |
| **14. Patients health status and medical-related factors** | | | | |
| **14.1 Physical or mental health** | | | | |
| *1* | *3* |  | *8* | *12* |
| Use of assistive devices (Dermody et al., 2018) | Premorbid independent state (Chan et al., 2019) |  | Patient condition (Dermody et al., 2018) | Patient having symptoms: weakness, fatigue, dyspnea, shortness of breath, pain (Brown et al., 2007) |
|  | Functional independent patients (De Klein et al., 2019) |  | Fall risk of patient* (p < .05) (Dermody et al., 2018) | Health-related vulnerability (Boltz et al., 2011) |
|  | Patients with low risk, e.g. patients who were small and lighter (Doherty-King et al., 2011) |  | Patients too sick (Dermody et al., 2017) | Acute ill patients (Doherty-King et al., 2011) |
|  |  |  | Impaired mobility of patient* (p < .05) (Dermody et al., 2018) | Patient having pain (Chan et al., 2019) |
|  |  |  | Use of assistive devices* (p < .05) (Dermody et al., 2018) | Patient with high risk, e.g. physically unstable, large in size, compromised cognitive functioning (Doherty-King et al., 2011) |
|  |  |  | Physical constraints or pain (Scheerman et al., 2020) | Patients too ill to move (Kirk et al., 2019; Pedersen et al., 2020) |
|  |  |  | Patient confusion (Said et al., 2021) | Patients acuity, e.g. delirum, dementia (Moore et al., 2014) |
|  |  |  | Medical factors, e.g. hypotension or pain (Said et al., 2021) | Depression or loneliness (Boltz et al., 2011) |
|  |  |  |  | Psychological barriers, e.g. patients fear of falling, newly diagnosed medical conditions, feeling of loneliness (Chan et al., 2019) |
|  |  |  |  | Patient's medical status (Haslam-Larmer et al., 2021) |
|  |  |  |  | Patient health status (Pavon et al., 2021) |
| **14.2 Treatment-related factors** | | | | |
| *1* | *3* |  | *0* | *7* |
| Presence of physical therapy orders* (p < .05) (Dermody et al., 2018) | Reject or change (bed rest) orders into activity (Doherty-King et al., 2011) |  |  | Presence of medical devices (De Klein et al., 2019) |
|  | Manage patient syndromes (Boltz et al., 2011) |  |  | Having intravenous line or urinary catheter (Brown et al., 2007) |
|  | Prevent complications (Doherty-King et al., 2011) |  |  | Patient is unfamiliar with medical devices (Chan et al., 2019) |
|  |  |  |  | Prolonged bed rest (Chan et al., 2019) |
|  |  |  |  | Complied with (bed rest) orders (Doherty-King et al., 2011) |
|  |  |  |  | Placement of patient care equipment or support, e.g. lines or tethers (Moore et al., 2014) |
|  |  |  |  | Patient physical restrictions (S. Lim et al., 2020) |
| **Institutional level** | | | | |
| **15. Physical environment** | | | | |
| **15.1 Space and location** | | | | |
| *0* | *9* |  | *0* | *9* |
|  | Design of physical spaces and objects, e.g. size, design, atmosphere and accessibility (Kirk et al., 2019) |  |  | Inadequate amount of space, equipment and furniture (Boltz et al., 2011) |
|  | Safe walking areas, lighting and flooring, and communal areas (Boltz et al., 2011) |  |  | Lack of chairs in patient rooms (Brown et al., 2007) |
|  | Adjustments in the hospital environment (De Klein et al., 2019) |  |  | Physical setup of patient room not encouraging mobility (Brown et al., 2007) |
|  | Enhance physical environment, e.g. patient room and ward (S.H. Lim et al., 2020) |  |  | Small patient rooms, lack of space, lack of dining and living rooms, objects in corridors (Kirk et al., 2019) |
|  | Space for walking in halls (Zisberg et al., 2018) |  |  | Intimidating environment (S. Lim et al., 2020) |
|  | Adjustments to patients rooms to make them more attractive for PA (Scheerman et al., 2020) |  |  | Lack of space (S. Lim et al., 2020; Moore et al., 2014; Tousignant-Laflamme et al., 2015) |
|  | Living room and walking routes (Scheerman et al., 2020) |  |  | Department's interior does not fit with mobility (Pedersen et al., 2020) |
|  | Increased amount of possibilities in the living room and lounge (Feenstra et al., 2021) |  |  |  |
|  | Placing the TV in the seating area instead of above the beds (Feenstra et al., 2021) |  |  |  |
| **16. Resources** | | | | |
| **16.1 Staffing** | | | | |
| *1* | *4* |  | *2* | *8* |
| Sufficient staffing ratio (Scheerman et al., 2020) | Availability of staff (Doherty-King et al., 2011) |  | Inadequate nurse to patient staffing (Dermody et al., 2017) | Staff shortage (Brown et al., 2007; S.H. Lim et al., 2020; Moore et al., 2014) |
|  | Presence of extra staff (Tousignant-Laflamme et al., 2015) |  | Searching for assistance from staff (Dermody et al., 2018) | Turnover of staffing (Kavanagh et al., 2019; Tousignant-Laflamme et al., 2015) |
|  | New position of allied health assistance (Mudge et al., 2015) |  |  | Fewer staffing in the evening (Kirk et al., 2019) |
|  | More staff, e.g. physiotherapists and medical students (Scheerman et al., 2020) |  |  | Staffing constraints (Boltz et al., 2011) |
|  |  |  |  | Staffing ratio (Scheerman et al., 2020) |
| **16.2 Time and competing priorities** | | | | |
| *0* | *2* |  | *8* | *19* |
|  | Reducing time issues (Andreasen et al., 2018) |  | Lack of time (Dermody et al., 2017; Zisberg et al., 2018; Hamilton et al., 2019) | Time constraints (Boltz et al., 2011; Brown et al., 2007; Chan et al., 2019; S.H. Lim et al., 2020) |
|  | Convenient timing (S. Lim et al., 2020) |  | Conflicting priorities (Dermody et al., 2018) | Lack of time (Moore et al., 2014; Tousignant-Laflamme et al., 2015; Pedersen et al., 2020) or limit time (De Klein et al., 2019) |
|  |  |  | Time constraints* of nurses with less experience (p = .048) (Dermody et al., 2017) | Competing HCP priorities (De Klein et al., 2019; S.H. Lim et al., 2020) |
|  |  |  | Workload (Dermody et al., 2017; Dermody et al., 2018) | Time and priority (Kirk et al., 2019) |
|  |  |  | Time constraints or low priority (Said et al., 2021) | Time pressure and tasks prioritizing (Andreasen et al., 2018) |
|  |  |  |  | Inconvenient timing (S. Lim et al., 2020) |
|  |  |  |  | Competing unit priorities (Moore et al., 2014) |
|  |  |  |  | Workload (Chan et al., 2019; Moore et al., 2014; Scheerman et al., 2020) |
|  |  |  |  | Available time (Pavon et al., 2021) |
|  |  |  |  | Lower priority of PA when being busy (Scheerman et al., 2020) |
| **16.3 Equipment** | | | | |
| *1* | *6* |  | *1* | *5* |
| Availability of equipment (Scheerman et al., 2020) | The right equipment (Boltz et al., 2011) |  | No proper equipment to mobilize patients (Zisberg et al., 2018) | Lack of ambulatory devices (Brown et al., 2007) |
|  | Provider incentives, e.g. equipment (Moore et al., 2014) |  |  | Patients are missing personal mobility aids from home (Moore et al., 2014) |
|  | Availability of material resources (Boltz et al., 2011; Doherty-King et al., 2011) |  |  | Lack of mobility equipment (Zisberg et al., 2018; Moore et al., 2014; Tousignant-Laflamme et al., 2015) |
|  | Equipment (Scheerman et al., 2020) |  |  |  |
|  | More tools to help patients become more active (Feenstra et al., 2021) |  |  |  |
| **16.4. Education and training** | | | | |
| *0* | *7* |  | *2* | *1* |
|  | Patient education about risks and steps to prevent (Boltz et al., 2011) |  | Lack of HCP training on how to safely mobilize patients (Dermody et al., 2017) | Little information about role of PA during admission (De Klein et al., 2019) |
|  | HCP training (Zisberg et al., 2018; Kavanagh et al., 2019) |  | Volunteer training (Babine et al., 2019) |  |
|  | Educational HCP meetings (Moore et al., 2014) |  |  |  |
|  | Distribution of printed educational materials for patients and HCP (Moore et al., 2014) |  |  |  |
|  | Reminders to patients and HCP (Moore et al., 2014) |  |  |  |
|  | Educational exhibits (Moore et al., 2014) |  |  |  |
| **16.5 Monitoring and documentation** | | | | |
| *0* | *5* |  | *0* | *3* |
|  | Evaluation strategy and monitoring (Kavanagh et al., 2019) |  |  | Lack of consistent system to document and monitor mobility (Moore et al., 2014) |
|  | Documentation techniques for tracking patients mobility status (Moore et al., 2014) |  |  | Unclear distance assessment (Zisberg et al., 2018) |
|  | Monitor patients progress (Doherty-King et al., 2011) |  |  | No reporting system (Zisberg et al., 2018) |
|  | Focus on measurable physical functioning (Van der Sluis et al., 2015) |  |  |  |
|  | Screening patients physical functioning before surgery (Van der Sluis et al., 2015) |  |  |  |
| **17. Organizational factors** | | | | |
| **17.1 Hospital routines and activities** | | | | |
| *0* | *9* |  | *0* | *7* |
|  | Initiation and coordination of physical therapy among HCPs (S.H. Lim et al., 2020) |  |  | Busy days at the unit (Doherty-King et al., 2011) |
|  | Activities: daily activity program (De Klein et al., 2019); meaningfull activities (Andreasen et al., 2018); social activities (Moore et al., 2014) |  |  | Busy clinical environment (S. Lim et al., 2020) |
|  | Integration program into usual care (Kavanagh et al., 2019) |  |  | Difficulty measuring patients function (Boltz et al., 2011) |
|  | Setting patient functional goals (Van der Sluis et al., 2015) |  |  | Lack of follow-through of HCP (Boltz et al., 2011) |
|  | Making ambulation visible on the ward for patients and staff (King et al., 2016) |  |  | Getting ready for discharge is unexpected (Doherty-King et al., 2011) |
|  | Setting patient ambulation goals (King et al., 2016) |  |  | Start ambulation is often during phase 'getting ready for discharge' with little time (Doherty-King et al., 2011) |
|  | Activity counseling (Scheerman et al., 2020) |  |  | Frequent patient transfers (S. Lim et al., 2020) |
| **17.2 Rules, regulations and policies** | | | | |
| *0* | *8* |  | *0* | *10* |
|  | Facility wide adoption of function-promoting philosophy (Boltz et al., 2011) |  |  | Fall prevention initiatives against PA (S.H. Lim et al., 2020) |
|  | Meaningful protocols (Boltz et al., 2011) |  |  | Hospital policies and protocols for zero falls (Chan et al., 2019) |
|  | Clear expectations of unit level with some accountability (Doherty-King et al., 2011) |  |  | Falls as quality indicators and mobility not (Zisberg et al., 2018) |
|  | Accountability of mobilizing patients (Doherty-King et al., 2011) |  |  | Limiting practices, e.g. mandatory wheelchair use (Boltz et al., 2011) |
|  | Alignment with institutional priorities (Kavanagh et al., 2019) |  |  | No standardized procedure for promoting PA (De Klein et al., 2019) |
|  | Audit and feedback (Moore et al., 2014) |  |  | No mobility protocols (Zisberg et al., 2018) |
|  | Budget to support proposed changes (Zisberg et al., 2018) |  |  | Facility-wide and unit-based expectations on patient mobilizing (Doherty-King et al., 2011) |
|  | Hospital focus on increasing patient awareness on importance of PA (Scheerman et al., 2020) |  |  | Formal document regarding patients’ mobility not familiar by staff (Zisberg et al., 2018) |
|  |  |  |  | Lack of accountability for HCP implementing the actionable recommendations (Moore et al., 2014) |
|  |  |  |  | Fall prevention is priority (Pavon et al., 2021) |

Abbreviations: RN = registered nurse; NA = nurse assistant; MD = medical doctor, *, statistical significant change p < 0,05.

^a^ Number of items according to subthemes
