# Supplementary material for: Facilitators and barriers to enhancing physical activity in older patients during acute hospital stay: a systematic review
Source: Int J Behav Nutr Phys Act. 2022 Jul 30;19:99. doi: 10.1186/s12966-022-01330-z (PMC9338465; doi:10.1186/s12966-022-01330-z)
Supplement: Supplementary file 8 — Additional file 8. Best-evidence synthesis classification. Table presenting an overview of the best-evidence synthesis classification for facilitators and/or barriers within quantitative studies. [file 12966_2022_1330_MOESM8_ESM.docx]

**Additional file 8.** Best-evidence synthesis classification for facilitators and/or barriers within quantitative studies.

| **Factor (facilitator or barrier)** | **From perspective^a^** | **Within subtheme** | **Explanation of Level of Evidence assessment^b^** | **Level of evidence** | **References** |
| --- | --- | --- | --- | --- | --- |
| Patients' unwillingness or refusal to move (barrier) | Patient | 1.1 Knowledge, awareness, attitude | 1 RCT high quality 1 RCT low quality | Moderate | Moreno et al., 2019;  Hamilton et al., 2019 |
| Rest or staying in bed would contribute to recovery (barrier) | Patient | 1.1 Knowledge, awareness, attitude | 1 MM non-randomized, low quality 1 MM quantitative descriptive, low quality | Insufficient | Zisberg et al., 2018;  Scheerman et al., 2021; |
| Age, e.g. older (barrier) or younger (facilitator) | Patient | 2.1 Patient personal factors | 4 Non-randomized studies, low quality | Insufficient | Agostini et al., 2014;  Resnick et al., 2015;  Ishikawa et al., 2020;  Zisberg et al., 2016 |
| Patients' fear, e.g. of infection, falling, missing a doctor's visit, or losing venous access (barrier); or less anxiety symptoms (facilitator) | Patient | 2.2 Emotional status | 1 RCT high quality 1 Non-randomized study, low quality | Limited | Moreno et al., 2019 Zisberg et al., 2016 |
| Higher baseline physical performance, or pre-fracture functional status score, or functional mobility, or physical activity level (facilitator); or lower preoperative physical activity level (barrier) | Patient | 2.4 Physical health | 2 Non-randomized studies, high quality 4 Non-randomized studies, low quality | Limited | McCullagh et al., 2020;  Said et al., 2020 Zisberg et al., 2016 Haslam-Larmer et al., 2021; Hartley et al., 2020 Agostini et al., 2014 |
| Having a hearing impairment (barrier) | Patient | 2.4 Physical health | 1 Non-randomized study, high quality | Insufficient | Chase et al., 2018 |
| Having symptoms, e.g. dyspnea, pain, dizziness, delirium (barrier) | Patient | 2.4 Physical health | 1 RCT high quality  2 Non-randomized study, high quality  1 Non-randomized studies, low quality  1 MM non-randomized, low quality  1 MM quantitative descriptive, low quality | Moderate | Moreno et al., 2019;  Said et al., 2021;  Haines et al., 2013;  S. Lim et al., 2020;  Agostini et al., 2014;  Scheerman et al., 2021 |
| Having lines or drains that hinder walking, e.g. intravenous medication or indwelling catheter (barrier) | Patient | 3.1 Presence of lines/attachments | 1 RCT high quality 2 Non-randomized studies, high quality 1 MM quantitative descriptive, low quality | Moderate | Moreno et al., 2019;  McCullagh et al., 2020;  Chua et al., 2017;  Scheerman et al., 2021 |
| Improving medical status or no acute complications (facilitator) | Patient | 3.2 Admitting diagnosis and illness severity | 2 Non-randomized studies, high quality | Limited | McCullagh et al., 2020;  Chua et al., 2017 |
| Non-emergency admit (barrier) | Patient | 3.2 Admitting diagnosis and illness severity | 1 Non-randomized study, high quality | Insufficient | Chase et al., 2017 |
| Lower illness severity (facilitator) or major illness severity (barrier) | Patient | 3.2 Admitting diagnosis and illness severity | 3 Non-randomized studies, low quality | Insufficient | Zisberg et al., 2016;  Hartley et al., 2020;  Fisher et al., 2011 |
| Absence of donor blood transfusion (facilitator) | Patient | 3.3 Treatment-related factors | 1 Non-randomized study, high quality | Insufficient | Chua et al., 2017 |
| Presence of bedrest order (barrier) | Patient | 3.3 Treatment-related factors | 1 Non-randomized study, high quality | Insufficient | McCullagh et al., 2020 |
| Longer length of stay (barrier) | Patient | 3.3 Treatment-related factors | 1 Non-randomized study, high quality | Insufficient | Chase et al., 2017 |
| Continues oxygen therapy (barrier) | Patient | 3.3 Treatment-related factors | 1 RCT high quality | Limited | Moreno et al., 2019 |
| Lack of companion encouragement, or patient alone (barrier) | Patient | 4.1 Patient - informal network | 1 RCT high quality 1 MM non-randomized, low quality | Limited | Moreno et al., 2019 Belala et al., 2019 |
| Lack of professional help (barrier), or presence of healthcare professional (facilitator) | Patient | 4.2 Patient - HCP | 1 RCT high quality 1 MM non-randomized, low quality | Limited | Moreno et al., 2019 Belala et al., 2019 |
| Lack of space (barrier) | Patient | 5.1 Space and location | 1 RCT high quality | Limited | Moreno et al., 2019 |
| Lack of equipment (barrier) | Patient | 6.3 Equipment | 1 RCT high quality 1 MM non-randomized, low quality | Limited | Moreno et al., 2019;  Scheerman et al., 2021 |
| Hospital site (facilitator) | Patient | 7.1 Hospital routines and activities | 1 Non-randomized study, high quality | Insufficient | Chua et al., 2017 |
| Patient not available (barrier) | Patient | 7.1 Hospital routines and activities | 1 RCT low quality 1 MM non-randomized, low quality | Insufficient | Hamilton et al., 2019;  S. Lim et al., 2020 |
| Wednesday (facilitator) | Patient | 7.2 Daytime or weekday | 1 Non-randomized study, high quality | Insufficient | McCullagh et al., 2020 |
| First day of admission (barrier) | Patient | 7.2 Daytime or weekday | 2 Non-randomized study, high quality 1 Non-randomized studies, low quality | Limited | McCullagh et al., 2020;  Haines et al., 2013;  Agostini et al., 2014 |
| Knowledge of PA (facilitator), or low knowledge of PA (barrier) | HCP | 10.1 | 1 Non-randomized study, low quality 1 MM non-randomized, low quality 1 MM quantitative descriptive, low quality | Insufficient | Dermody et al., 2018;  Zisberg et al., 2018;  Scheerman et al., 2020 |
| Risk of staff or patient injury while performing tasks (barrier) | HCP | 10.2 Patient safety concerns | 1 Non-randomized study, high quality 1 Non-randomized study, low quality 1 Quantitative descriptive study, low quality | Insufficient | Said et al., 2021;  Dermody et al., 2017;  Babine et al., 2019 |
| Patients declined to mobilize (barrier) | HCP | 12.2 Patient-HCP | 1 Non-randomized study, high quality | Insufficient | Said et al., 2021 |
| Having symptoms, e.g. hypotension, pain, or patient confusion (barrier) | HCP | 14.1 Physical or mental health | 1 Non-randomized study, high quality 2 Non-randomized studies, low quality 1 MM quantitative descriptive, low quality | Insufficient | Said et al., 2021;  Dermody et al., 2017; Dermody et al., 2018; Scheerman et al., 2020 |
| Lack of time or time constraints (barrier) | HCP | 16.2 Time and competing priorities | 1 RCT low quality 1 Non-randomized study, high quality 1 Non-randomized study, low quality 1 MM non-randomized, low quality | Limited | Hamilton et al., 2019;  Said et al., 2021;  Dermody et al., 2017;  Zisberg et al., 2018 |
| Workload (barrier) | HCP | 16.2 Time and competing priorities | 2 Non-randomized studies, low quality | Insufficient | Dermody et al., 2017;  Dermody et al., 2018 |
| Available equipment (facilitator), or no proper equipment to mobilize patients (barrier) | HCP | 16.3 Equipment | 1 MM non-randomized, low quality 1 MM quantitative descriptive, low quality | Insufficient | Zisberg et al., 2018;  Scheerman et al., 2020 |

Abbreviations: MM = mixed-methods study design; high quality study = MMAT score 4 or 5 on criteria set 1) randomized controlled trials, 2) non-randomized studies, or 3) quantitative descriptive studies, or MMAT score 4 or 5 on quantitative part of mixed-methods study design; low quality study = MMAT score 0, 1, 2, 3 on criteria set 1) randomized controlled trials, 2) non-randomized studies, or 3) quantitative descriptive studies, or MMAT score 4 or 5 on quantitative part of mixed-methods study design.

^a^ Patient, informal caregiver, or HCP

^b^ based on study quality, study design type, quantity, and consistency of findings
